# MTX2 facilitates PKM2 tetramerization to promote cardiac glucose metabolism and protects the heart against ischemia/reperfusion injury

**DOI:** 10.7150/thno.110162

**Published:** 2025-06-09

**Authors:** Yueyang Li, Yu Li, Yang Li, Yufan Jiang, Miao Wang, Mingyi Wang, Jie Liu, Mingrui Ma, Xiaofeng Zhai, Li Yi, Tao Chen, Zhenyu Xiong, Yundai Chen

**Affiliations:** 1 Department of Cardiology, the Sixth Medical Centre, Chinese PLA General Hospital, Beijing, 100048, China.; 2 Department of Cardiology, the First Medical Centre, Chinese PLA General Hospital, Beijing, 100853, China.; 3 Medical School of Chinese PLA, Chinese PLA General Hospital, Beijing, 100853, China.; 4 The Sixth Medical Centre, Chinese PLA General Hospital, Beijing, 100048, China.

**Keywords:** myocardial ischemia/reperfusion injury, metaxin 2, mitochondria, pyruvate kinase M2, metabolic homeostasis.

## Abstract

**Rationale:** Myocardial ischemia reperfusion (I/R) injury is a major cause of adverse outcomes following revascularization therapy. Although alterations in metabolic activities during reperfusion have been implicated, the molecular mechanisms underlying the pathogenesis of I/R injury remain elusive. Metaxin 2 (MTX2), initially identified as a core component of protein import complexes, has recently been characterized in diverse cellular functions. Nevertheless, its involvement in myocardial I/R injury has yet to be fully elucidated. In this study, we aim to evaluate the role and the underlying mechanism of MTX2 in I/R injury.

**Methods:** The myocardial I/R model was established, and the protein levels of MTX2 were determined at different time points following coronary occlusion. Loss-of-function and gain-of-function strategies were applied via genetic ablation or intra-myocardial adenovirus injection to ascertain the role of MTX2 in myocardial I/R injury. RNA sequencing, seahorse metabolic analysis, and mass spectrometry were conducted to uncover the underlying molecular mechanisms.

**Results:** We observed that the expression of MTX2 was significantly decreased in I/R hearts. Tamoxifen-induced cardiomyocyte-specific deletion of *Mtx2* led to aggravated myocardial I/R injury, resulting in impaired cardiac oxidative phosphorylation and glycolysis. Mechanistically, dimeric PKM2, a less active pyruvate kinase form compared with tetrameric PKM2, was found to be dramatically accumulated in *Mtx2* deficiency mice after myocardial I/R surgery. The TOM37 domain of MTX2 interacted directly with PKM2 to promote PKM2 tetramerization, thereby modulating glucose metabolic flux. Pharmacological activation of PKM2 by a small-molecule PKM2 activator, TEPP-46, rescued the metabolic and functional outcomes of I/R in *Mtx2* deficiency mice.

**Conclusions:** Our results identified, for the first time, a cardioprotective role of MTX2 in modulating cardiac glucose metabolism by facilitating PKM2 tetramerization. Targeting metabolic homeostasis by restoring MTX2 might be a promising therapeutic strategy to mitigate myocardial I/R injury.

## Introduction

Ischemic heart disease is among the leading causes of death in developed as well as developing countries 1. Despite prompt and comprehensive revascularization by percutaneous coronary intervention, this reperfusion process paradoxically causes myocardial ischemia/ reperfusion (I/R) injury, pathological manifestations of which include enlarged myocardial necrotic areas, energy depletion, and ultimately contraction dysfunction 2. To date, effective pharmacological interventions are yet to be developed to meet current clinical needs 3. The heart is one of the most prominent energy-consuming organs and, thus, cardiac substrate metabolism is instrumental in maintaining the normal structure and function of the heart. Energy metabolic disturbance, in particular, glucose and fatty acid (FA) metabolism, has been recognized as a critical determinant of I/R damage 4. However, the upstream event contributing to impaired substrate metabolism in the I/R hearts remains inadequately characterized.

As central hubs of cardiac ATP production, mitochondria are instrumental in maintaining metabolic balance during I/R. Thus, targeting molecules primarily located in mitochondria shows promise for intervening I/R-induced ATP defect and metabolic disturbance 5-8. The Sorting and Assembly Machinery (SAM) complex, composed of metaxin 1 (MTX1), metaxin 2 (MTX2) and SAM50, have emerged as promising therapeutic targets through its coordinated governance of mitochondrial proteostasis, dynamic morphological remodeling, and cristae architecture preservation 9-12. However, evidence for their functional implications in cardiovascular diseases remains strikingly limited. MTX2, also known as SAM37, was initially reported to collaborate with MTX1 and SAM50 for the accurate integration of β-barrel proteins into the mitochondrial outer membrane, thus ensuring proper assembly of essential proteins such as VDAC channels 13,14. Unlike MTX1 and SAM50, MTX2 lacks a C-terminal mitochondrial outer membrane signal-anchor domain. Instead, MTX2 is oriented towards the cytosolic compartment of the mitochondrial outer membrane through its interaction with membrane-bound MTX1 15,16, indicating a potential role as a mediator of mitochondrial-cytosolic crosstalk beyond its established transport function. Recent research has further expanded its role as a direct regulator of TNF-α induced apoptosis 16,17. Furthermore, *Mtx2* deficiency can lead to a spectrum of adverse effects, such as reduced oxidative phosphorylation, excessive mitochondrial fission, and altered nuclear morphology 12,18. Despite these findings, it remains unclear whether MTX2 plays a role in cardiac energy metabolism and myocardial I/R injury. Furthermore, its subcellular and molecular mechanisms responsible for MTX2-related cardiac regulation have not been identified.

In this study, we generated mice with an inducible cardiomyocyte-specific deletion of *Mtx2* using α-MHC-MerCreMer. Through the implementation of loss- and gain-of-function strategies, we sought to explore how MTX2 regulates cardiac metabolism in the pathogenesis of I/R injury and its therapeutic potential. Considering the important role of MTX2 in cardiac oxidative phosphorylation (OXPHOS) and glycolysis, these results provide proof of concept that targeting MTX2 might mitigate myocardial I/R injury and other cardiac diseases characterized by metabolic defects.

## Methods

Extended methods sections are available in the Supplemental Material.

### Animals

Animal studies were performed according to the National Institutes of Health Guidelines for the Care and Use of Laboratory Animals (NIH publication No. 85-23, revised 2011). Animal protocols and performances were approved by the Institutional Animal Care and Use Committee of PLA General Hospital. Euthanasia was performed in accordance with the American Veterinary Medical Association (AVMA) Guidelines for the Euthanasia of Animals (2020) and with the approval of local animal welfare committees.

Adult male C57BL/6J mice were purchased from Model Organisms Center, Inc. (Shanghai, China). Mice were anesthetized by inhalation of 2% isoflurane in oxygen throughout the cardiac I/R surgery, intra-myocardial injection of adenovirus, echocardiography and heart excision for adult cardiomyocyte isolation. Finally, mice were euthanized in their home cage via carbon dioxide inhalation. Carbon dioxide was introduced into the cage at a rate of 3 L/min, added to the existing air in the chamber, until the cessation of respiration in the mice was observed. Mice were then remained in cages for an additional 3 min. Cervical dislocation was performed thereafter to ensure death. The hearts were either stored at -80 °C for subsequent protein extraction or immersed in 4% paraformaldehyde for histological analyses.

### Data availability

All supporting data in the study are available from the corresponding author on reasonable request. RNA sequencing data of this study have been uploaded to the National Center for Biotechnology Information (NCBI) BioProject database under the accession number PRJNA1136562.

### Statistical analysis

All values were presented as mean ± SD. Data were analyzed using GraphPad Prism 8 statistic software. The normality of the data was assessed using the Shapiro-Wilk test. For normally distributed data, Student's t-test was carried out to compare differences between two groups, and one-way or two-way ANOVA followed by Tukey post-hoc test was used for multiple group comparisons. For non-normally distributed data, Mann-Whitney U test was used for two-group comparisons, and Kruskal-Wallis test with Dunn multiple comparison test was applied for comparisons involving multiple groups. P values < 0.05 were considered statistically significant.

## Results

### MTX2 is downregulated in myocardial I/R hearts

**Figure 1:**
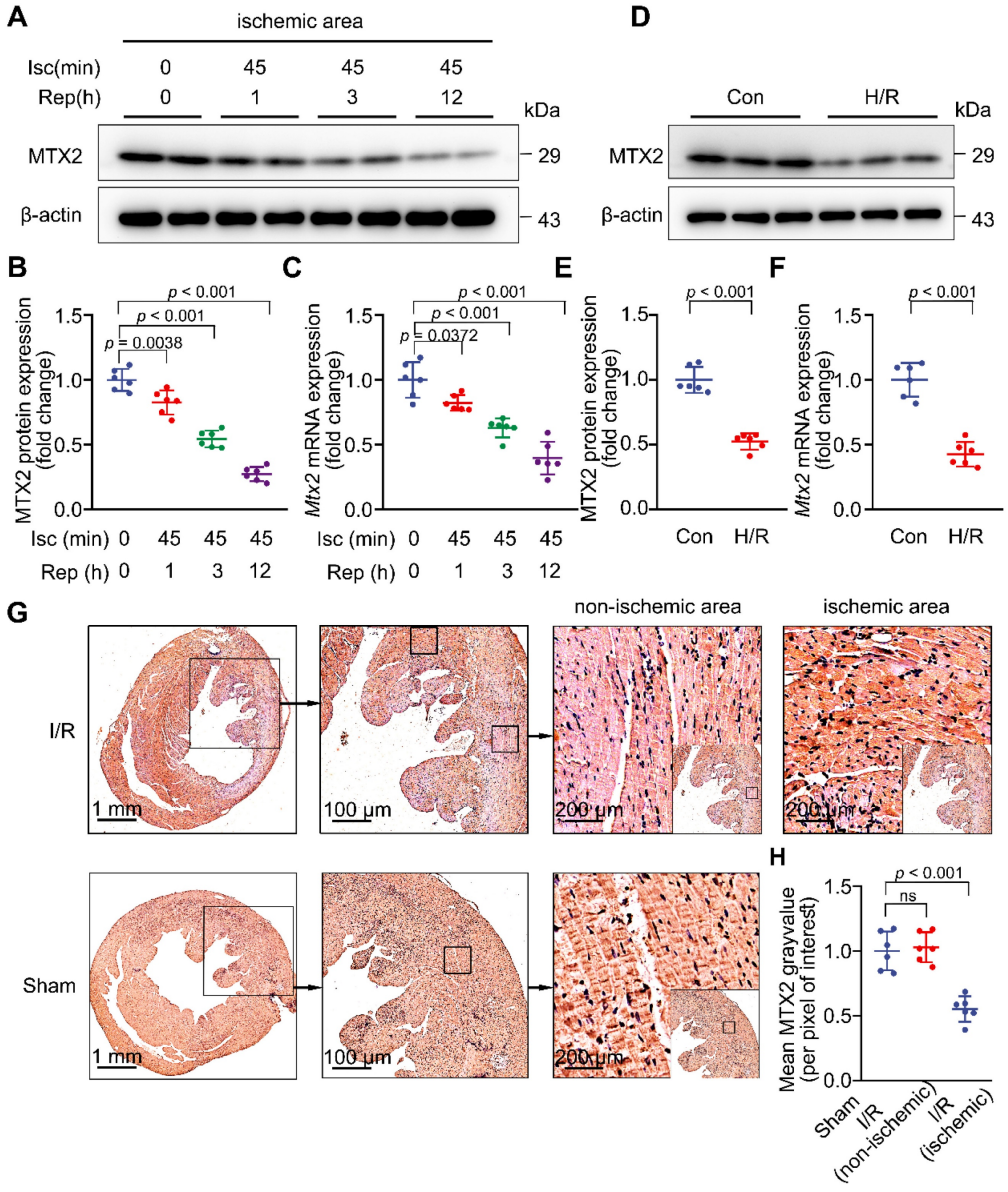
** MTX2 is downregulated in myocardial I/R hearts.** *In vivo*, 10-week WT mice were subjected to 45-min ischemia followed by reperfusion at different time points. *In vitro*, neonatal rat ventricular cardiomyocytes (NRVMs) were isolated and treated with 9-h of hypoxia and 3-h of reoxygenation (H/R) to simulate the ischemia/reperfusion (I/R) injury. **(A-B)** Western blot and associated quantification of MTX2 protein levels in ischemic myocardial tissue at the indicated time points after I/R induction (n = 6). **(C)** Quantitative real-time polymerase chain reaction (qPCR) analysis of heart *Mtx2* levels in ischemic myocardial tissue at the indicated time points after I/R induction (n = 6). **(D-E)** Western blot and associated quantification of MTX2 protein levels in reoxygenated cardiomyocytes (n = 6). **(F)** qPCR analysis of *Mtx2* mRNA levels in cardiomyocytes under H/R condition (n = 6). **(G-H)** Representative immunohistochemical staining of MTX2 in ischemic and non-ischemic myocardial tissue after 3-h reperfusion and its associated quantification (n = 6. Scale bars: 1 mm (left row), 100 μm (middle row), 200 μm (right row). **(B, C, H)** Data were analyzed by one-way ANOVA followed by Tukey post-hoc test.** (E, F)** Data were analyzed by Two-tailed unpaired Student t-test. All values are presented as mean ± SD. ns, not significant; MTX2, metaxin 2; Isc, ischemia; Rep, reperfusion; I/R, ischemia/reperfusion; H/R, hypoxia/reoxygenation.

To investigate whether MTX2 plays a role in I/R injury, we first examined MTX2 protein and mRNA expression in adult mouse hearts at different time points of reperfusion. We found that MTX2 was abundantly expressed in the heart but significantly downregulated in response to 45-min ischemia followed by 1-h reperfusion. MTX2 protein was further decreased in the ischemic hearts as a consequence of 3-h and 12-h reperfusion (Figure 1A-1B). The protein expression pattern of MTX2 mirrored its mRNA pattern during myocardial reperfusion phase (Figure 1C). Moreover, we examined MTX2 protein and mRNA levels in non-ischemic myocardium after I/R surgery. By contrast, there was no statistically significant difference in MTX2 expression between non-ischemic myocardium and its sham control (Figure S1A-C). Consistently, declined cardiac MTX2 expression in ischemic myocardium was corroborated by immunohistochemical staining, in comparison to the sham-operated controls and non-ischemic myocardium (Figure 1G-H). Next, neonatal rat ventricular cardiomyocytes (NRVMs) were isolated and subjected to H/R attack. We found that MTX2 protein and mRNA expressions were significantly decreased in cardiomyocytes after H/R stimulation *in vitro* (Figure 1D-1F). However, MTX2 expression was not significantly changed at both protein and mRNA levels between the Con and H/R groups in both fibroblasts and endothelial cells (Figure S2A-F), suggesting that MTX2 expression was only decreased in myocardial cells in response to I/R attack. Taken together, given the decrease of MTX2 at both transcriptional and translational levels, a causative relationship between MTX2 and I/R insult is strongly indicated.

### Knockdown of *Mtx2* exacerbates cardiac I/R injury

To mimic the effects of MTX2 reduction observed in I/R mice and further gain insight into the role of MTX2 in myocardial I/R injury, cardiac-specific *Mtx2* knockout mice (cKO) were created using tamoxifen-inducible Cre recombinase system. *Mtx2^flox/flox^* mice were created by generating a floxed allele of *Mtx2* with exon 3 flanked by loxp sites. These mice were then crossed with α-MHC-MerCreMer mice (Figure S3A). The genotypes of the offspring mice were verified and *Mtx2^flox/flox^/*MerCreMer mice were injected with tamoxifen (40 mg/kg/day) for 5 days to induce specific deletion of the *Mtx2* gene in cardiomyocytes. Age-matched MerCreMer mice without the floxed *Mtx2* allele were administered an equivalent dose of tamoxifen and served as controls (Ctrl) throughout the entire experiment. After a 2-week washing-out period, the knockout efficiency of *Mtx2* in cKO mice was examined (Figure S3B). The genotype of cKO mice was confirmed by polymerase chain reaction (PCR) (Figure S3C). Moreover, the protein level of MTX2 was significantly decreased in the cKO group compared with Ctrl mice, validating the effective deletion of the *Mtx2* gene in cardiomyocytes after tamoxifen induction (Figure S3D-E).

**Figure 2:**
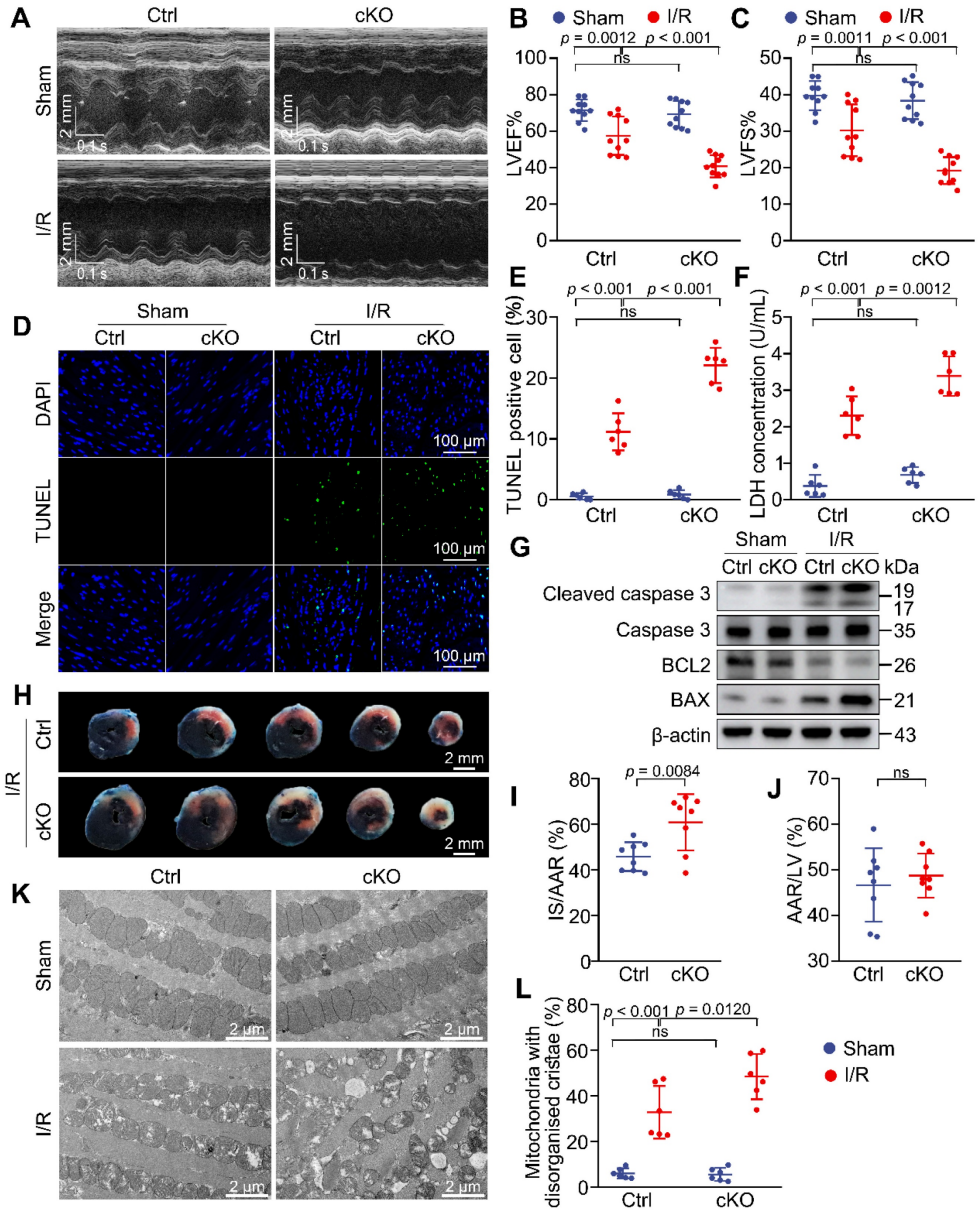
** Knockdown of *Mtx2* exacerbates cardiac I/R injury.** 10-week control (Ctrl) mice or *Mtx2* cKO (cKO) mice were subjected to sham or I/R surgery. **(A-C)** Representative M-Mode echocardiographic images and measurements of LV ejection fraction and LV fractional shortening at 24 h after reperfusion in the indicated groups (n = 10). **(D-E)** Representative cardiac apoptosis detected by TUNEL staining after 3-h reperfusion and TUNEL-positive cardiomyocyte quantification. Green: TUNEL-positive apoptotic nuclei; Blue: DAPI-stained total nuclei. (n = 6). Scale bar: 100 μm. **(F)** Cardiac damage by LDH release assay after 3-h reperfusion (n = 6). **(G)** Western blot of cleaved caspase 3, total caspase 3, BAX and BCL2 after 3-h reperfusion (n = 6). **(H-J)** Representative heart slices stained by Evans blue and tetrazolium chloride (TTC) after 24-h reperfusion. The infarcted size (IS, white), areas of the left ventricle (LV, red plus white plus blue), and areas at risk (AAR, red plus white) were calculated (n = 8). Scale bar: 2 mm. **(K-L)** Representative transmission electron microscopic (TEM) images of mitochondria in Ctrl or cKO mice subjected to sham or I/R surgery (magnification × 6000 [6 K]) (n=6). **(B, C, E, F, L)** Data were analyzed by two-way ANOVA, followed by a Tukey post-hoc test. (I, J) Data were analyzed by Two-tailed unpaired Student t-test. All values are presented as mean ± SD. ns, not significant; MTX2, metaxin 2; I/R, ischemia/reperfusion; cKO, cardiac knockout; Ctrl, control.

To assess the effects of *Mtx2* knockout on cardiac function, echocardiography was performed on cKO and their Ctrl littermates with sham or I/R surgery. In sham-operated animals, cardiac function was comparable between the Ctrl and cKO groups. However, 24 h post-I/R procedure, cKO mice showed aggravated cardiac dysfunction compared with the Ctrl group, as indicated by a marked decline in LV ejection fractions (LVEF) and fractional shortenings (LVFS), as well as a significant increase in LV end-diastolic diameter (LVEDD) and LV end-systolic diameter (LVESD) (Figure 2A-C, Table S1). Moreover, cardiac apoptosis was significantly increased in cKO mice after I/R attack, as evidenced by cleaved caspase-3/caspase-3 ratio, BAX/BCL2 ratio and TUNEL/DAPI double staining (Figure 2D, 2E, 2G and Figure S4). In agreement with these findings, *Mtx2*-deficient hearts showed a remarkable elevation of cardiomyocyte injury measured by LDH release (Figure 2F). Compared with the Ctrl group, the cKO hearts exhibited a significantly enlarged I/R-induced infarct size (Figure 2H-J). Additionally, *Mtx2* deletion aggravated I/R-induced mitochondria cristae disorder and the appearance of vacuoles (Figure 2K-L). Collectively, these *in vivo* molecular, structural, and functional experiments show that cardiac-specific deletion of *Mtx2* detrimentally affects the outcome of I/R insult.

### MTX2 is required for maintaining glucose metabolism in I/R hearts

**Figure 3:**
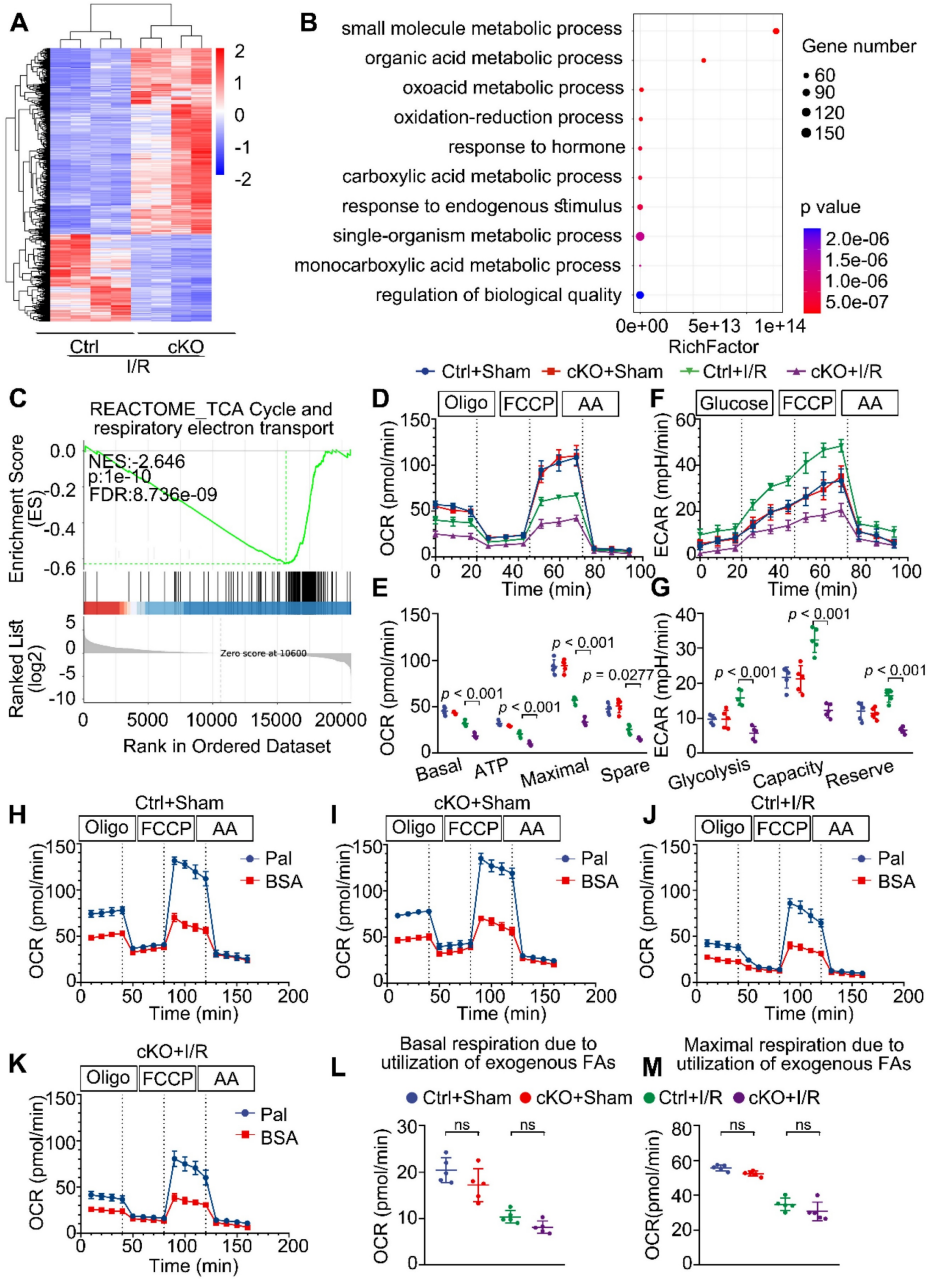
** MTX2 is required for maintaining glucose metabolism in I/R hearts. (A)** Cluster analysis of differentially expressed genes determined by RNA seq of I/R hearts in Ctrl and cKO mice (n = 4). **(B)** Top 10 Gene ontology (GO) pathways that were downregulated in heart tissue of cKO mice compared with Cre controls after I/R injury (n = 4). **(C)** Gene set enrichment analysis (GSEA) of RNA-seq data reveals *Mtx2* deletion is negatively correlated with TCA cycle and respiratory electron transport compared to Ctrl mice (n = 4). **(D)** OCR curve measured using Seahorse flux analyzer in adult cardiomyocytes isolated from post-I/R injury Ctrl and cKO mice (n = 5). **(E)** Quantification of basal respiration, maximal respiration, ATP production, and spare capacity according to instruction (n = 5). **(F)** ECAR curve measured using Seahorse flux analyzer in adult cardiomyocytes isolated from post-I/R injury Ctrl and cKO mice (n = 5). **(G)** Quantification of glycolysis, glycolytic capacity, and glycolytic reserve according to instruction (n = 5). **(H-K)** Fatty acid oxidation (FAO) curve measured using Seahorse flux analyzer in adult cardiomyocytes isolated from post-I/R injury Ctrl and cKO mice (n = 5). **(L)** Quantification of basal respiration due to exogenous FAs according to instruction (n = 5). **(M)** Quantification of maximal respiration due to exogenous FAs according to instruction (n = 5). Data were analyzed by two-way ANOVA, followed by a Tukey post-hoc test. All values are presented as mean ± SD. ns, not significant; MTX2, metaxin 2; I/R, ischemia/reperfusion; cKO, cardiac knockout; Ctrl, control.

To explore the molecular mechanisms responsible for the negative consequences of *Mtx2* deletion, we performed RNA sequencing (RNA-Seq) of cKO hearts subjected to sham or I/R operation. RNA-seq analysis revealed 2032 differentially expressed genes between Ctrl + I/R and cKO + I/R samples (Figure 3A). Gene Ontology (GO) enrichment analysis of the downregulated genes in cKO hearts identified significant enrichment of genes related to substrate metabolism (Figure 3B). Particularly noteworthy was the gene set enrichment analysis (GSEA) which highlighted the TCA cycle and respiratory electron transport as among the most significantly downregulated terms in the cKO + I/R group (Figure 3C).

To confirm the effect of MTX2 on substrate metabolism, we tested the metabolic phenotypes of glucose oxidation, glycolysis, and fatty acid oxidation in adult mouse ventricular myocytes isolated from post-I/R injury Ctrl and cKO mice through utilizing the Seahorse flux analyzer. As shown in Figure 3D-E, there were no apparent differences in the energy metabolic phenotypes between Ctrl and cKO mice in the absence of I/R attack. However, upon I/R exposure, cKO hearts exhibited an approximately 50% reduction in glucose oxidation, as calculated by basal and maximal OCRs, ATP production, and spare respiratory capacity. Moreover, *Mtx2* deficiency decreased glycolysis at both basal and maximal levels in cardiomyocytes under I/R condition (Figure 3F-G). Furthermore, when exogenous BSA or palmitate-BSA was added as an energy substrate, a slight decrease in both basal and maximal respiration due to utilization of exogenous palmitate-BSA was observed in cKO myocytes but with no significant difference (Figure 3H-M). These observations were consistent with the qPCR analysis of key glucose and fatty acid metabolic enzymes in Ctrl and cKO hearts under I/R attack (Figure S5A-C). Taken together, these results suggest that *Mtx2* deletion leads to severe impairment in cardiac OXPHOS activity and glycolytic flux under I/R attack.

### Identification of the kinase PKM2 (Pyruvate kinase M2) as an MTX2-interacting protein

**Figure 4:**
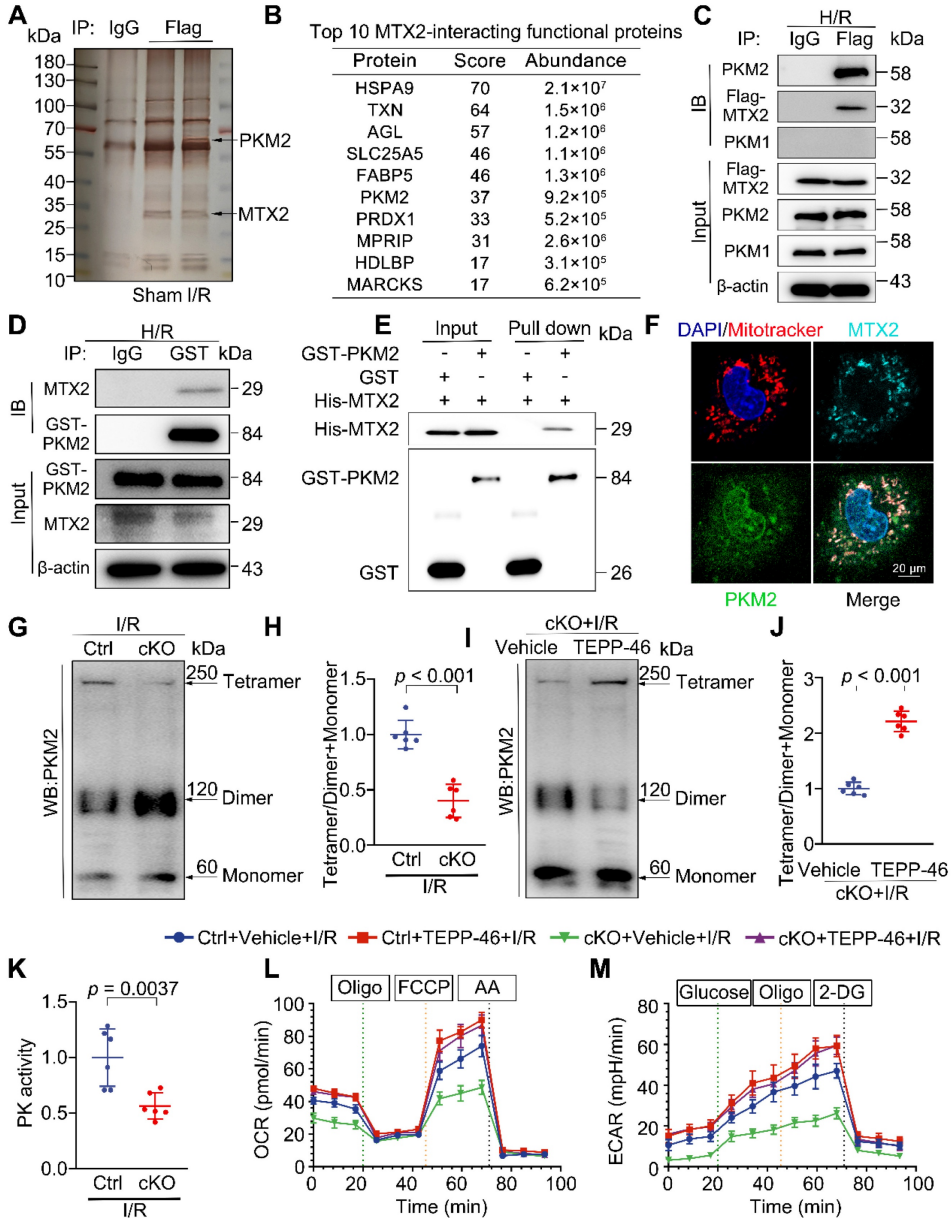
** MTX2 directly interacts with PKM2 to facilitate its tetramer formation hence promoting glucose metabolism. (A)** Mice were received intra-myocardial injection with Flag-tagged Ad-*Mtx2* 7 days before sham or I/R operation. The heart lysates were immunoprecipitated with an anti-Flag antibody or IgG (negative control antibody). The eluates were resolved on SDS-PAGE and silver stained. **(B)** Top 10 MTX2-interacting functional proteins from mass spectrometry analysis of peptides pulled down using anti-FLAG antibody. **(C)** Co-immunoprecipitation of overexpressed Flag-*Mtx2* and endogenous PKM1 and PKM2 in NRVMs. Lysates were immunoprecipitated with the anti-Flag antibody and immunoblotted with the indicated antibodies (n = 6). **(D)** Co-immunoprecipitation of overexpressed GST-PKM2 and endogenous MTX2 in NRVMs. Lysates were immunoprecipitated with the anti-GST antibody and immunoblotted with the indicated antibodies (n = 6). **(E)** Glutathione S-transferase (GST) pull-down assays with recombinant purified GST-PKM2 and His-MTX2. **(F)** Representative confocal microscopy images of immunofluorescence staining of MTX2, PKM2, Mitotracker and DAPI (4',6-diamidino-2-phenylindole) in NRVMs (n = 6). Scale bar, 20 μm. **(G-H)** Representative Western blot image and associated quantification of cardiac PKM2 oligomerization from Ctrl and cKO mice after 3-h reperfusion (n = 6). **(I-J)** Representative Western blot image and associated quantification of cardiac PKM2 oligomerization from cKO mice treated with TEPP-46 or vehicle 15 min before I/R surgery (n = 6). **(K)** PK activity in Ctrl and cKO mice under I/R attack (n = 6). **(L-M)** Ctrl or cKO mice were intraperitoneally injected with TEPP-46 15 min before I/R surgery. After 3-h reperfusion, adult cardiomyocytes were isolated for OCR and ECAR determination using a seahorse flux analyzer. **(L)** OCR curve of adult cardiomyocytes isolated from Ctrl and cKO mice treated with vehicle or TEPP-46 in the presence of I/R attack (n = 5). **(M)** ECAR curve of adult cardiomyocytes isolated from Ctrl and cKO mice treated with vehicle or TEPP-46 in the presence of I/R attack (n = 5). Data were analyzed by Two-tailed unpaired Student t-test. All values are presented as mean ± SD. ns, not significant; MTX2, metaxin 2; PKM2, pyruvate kinase M2; H/R, hypoxia/reoxygenation; I/R, ischemia/reperfusion; cKO, cardiac knockout; Ctrl, control.

To further search for the molecular mechanism of MTX2 regulating glucose metabolism, co-immunoprecipitation (Co-IP) was undertaken in heart tissues from mice with an intra-myocardial injection of flag-tagged MTX2. The IgG immunoprecipitation group served as the negative control. After electrophoresis, silver staining was performed. As shown in Figure 4A, differential spots were observed between samples under sham or I/R. The candidate MTX2-interacting proteins were detected by mass spectrometry (MS) in response to I/R treatment (Figure S6A). The top 10 MTX2-interacting functional proteins were listed in Figure 4B. We noticed PKM among the interacting proteins of MTX2, a key enzyme responsible for the irreversible conversion of phosphoenolpyruvate (PEP) to pyruvate to promote glycolytic flux or OXPHOS (Figure S6B). In light of the suppression of mitochondrial respiration and glycolytic flux, this interaction was quite intriguing and aligned with our disturbed metabolic phenotype. Next, the physical interaction of PKM with MTX2 in Ad-Flag-*Mtx2* infected NRVMs was validated by immunoblotting after immunoprecipitation. PKM1 and PKM2 are two alternative splice isoforms encoded by the same *Pkm* gene and differ only by alternative mRNA splicing 19. PKM2 was highly expressed in embryonic or stressed hearts, but displayed minimal expression in healthy adult hearts 20. In contrast, PKM1 is abundantly expressed in normal adult cardiomyocytes 21. As shown in Figure 4C, PKM2, rather than PKM1, was efficiently coimmunoprecipitated with MTX2, which was consistent with the aforementioned silver staining result of the band between 70 kDa and 55 kDa. We further substantiated the immunoprecipitation of MTX2 with PKM2 (Figure 4D). GST (glutathione S-transferase) pull-down assays were performed with purified recombinant GST-PKM2 and polyhistidine-MTX2, revealing a direct binding between the two proteins (Figure 4E). Consistently, the endogenous interaction of MTX2 with PKM2 was verified by immunofluorescence (Figure 4F).

Given that PKM2 might be a potential target binding protein of MTX2 in NRVMs, we next sought to investigate whether the mRNA or protein level of PKM2 was changed in *Mtx2*-deficient hearts. The expression of PKM2 was scarcely detectable under sham surgery and was significantly induced in response to I/R attack (Figure S7A-B). As shown in Figure S8A-B, there was no significant difference in the protein and mRNA levels of PKM2 between the Ctrl and cKO groups following I/R exposure. However, compared with the control group, the cKO hearts showed a notable decrease in PK activity in response to I/R challenge, indicating that MTX2 modulates PKM2 activity without affecting *Pkm2* transcription (Figure 4K).

### MTX2 modulates PKM2 tetramer formation to promote glucose metabolism

PKM2 has been found to primarily exist in two multimeric forms in tumor cells: an enzymatically active tetrameric form and a nearly inactive dimeric form at physiological concentrations of PEP 22. To identify the mechanism underlying MTX2's regulation of PKM2 activity, cross-linking studies were performed. As shown in Figure 4G-H, compared with the control hearts, the level of PKM2 tetramer was lower and the inactive dimer form was accumulated in the *Mtx2* knockout hearts after I/R attack. This was consistent with the observed reduction in PK activity and the overall efficiency of glucose metabolism. We next sought to find a small molecule PKM2 activator capable of reversing the clinical phenotypes and metabolic abnormalities due to reduced PKM2 activity caused by *Mtx2* deletion. TEPP-46 has been extensively characterized as a specific activator of PKM2, facilitating the conversion of PKM2 dimers to tetramers 23-25. We observed that compared with the cKO group, TEPP-46 administration successfully upregulated the tetramer form of PKM2, as well as the ratio of the tetramer to dimer + monomer forms in the I/R hearts (Figure 4I-J). We next asked whether declined PKM2 tetramerization was responsible for the metabolic dysfunction in the hearts of cKO mice. TEPP-46 was administered via intraperitoneal injection 15 min before I/R surgery. OCR and ECAR in adult cardiomyocytes isolated from Ctrl or cKO mice with TEPP-46 treatment were determined via a seahorse metabolic flux analyzer after I/R surgery. As shown in Figure 4L-M and Figure S9A-G, the reduced level of glycolysis and oxidative phosphorylation caused by *Mtx2* deletion was effectively rescued by TEPP-46. Importantly, the cKO and Ctrl mice pre-treated with TEPP-46 before I/R surgery exhibited comparable measurements of glucose oxidation and glycolysis, which suggests that activating PKM2 is sufficient to rescue the metabolic dysfunction caused by *Mtx2* knockout. Together, these data further support that MTX2 regulates cardiac glucose metabolism by interfering with the dimer/tetramer equilibrium of PKM2.

### MTX2 regulates PKM2 tetramerization via interacting with PKM2 through its TOM37 domain

**Figure 5:**
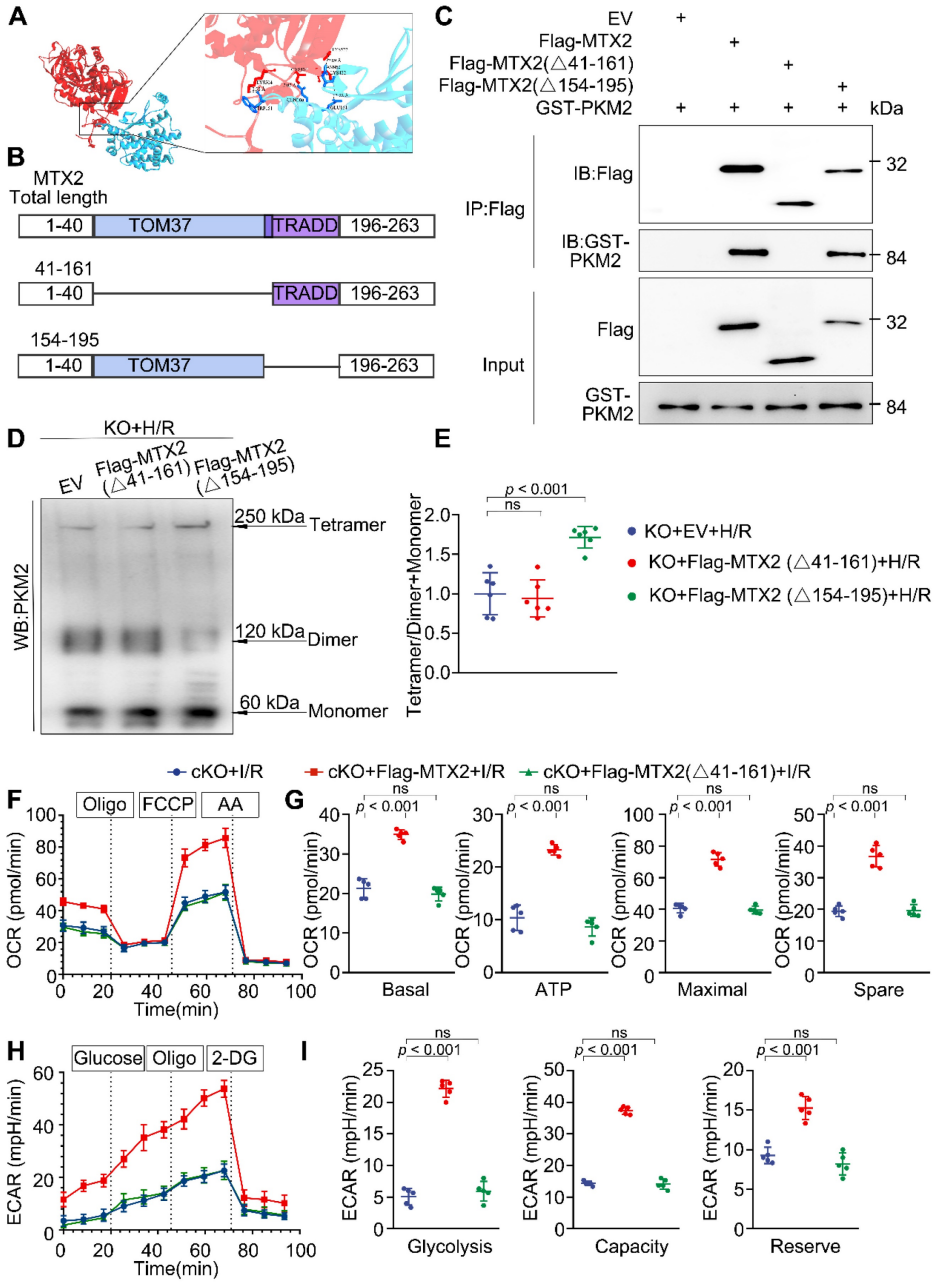
** MTX2 regulates PKM2 tetramerization via interacting with PKM2 through its TOM37 domain. (A)** Structure-based representation of MTX2 (blue)-PKM2 (red) protein binding model. **(B)** Construction of wild-type (total length) and truncated mutants of MTX2. **(C)** *Mtx2* KO H9C2 cells were transfected with Flag-tagged wild-type (total length) MTX2 and its truncated mutants. Whole-cell lysates were immunoprecipitated with anti-FLAG antibody and then immunoblotted with indicated proteins. **(D-E)** Representative Western blot image and associated quantification of cardiac PKM2 oligomerization from *Mtx2* KO H9C2 cells overexpressing MTX2 mutants before H/R attack (n = 6). **(F)** OCR curve of adult cardiomyocytes isolated from cKO mice overexpressing Flag-tagged adenovirus carrying full-length MTX2 or MTX2 (△41-161 AA) in the presence of I/R attack (n = 5). **(G)** Quantification of basal respiration, maximal respiration, ATP production, and spare capacity according to instruction (n = 5). **(H)** ECAR curve of adult cardiomyocytes isolated from cKO mice overexpressing Flag-tagged adenovirus carrying full-length MTX2 or MTX2 (△41-161 AA) in the presence of I/R attack (n = 5). **(I)** Quantification of glycolysis, glycolytic capacity, and glycolytic reserve according to instruction (n = 5). Data were analyzed by one-way ANOVA followed by Tukey post-hoc test. All values are presented as mean ± SD. ns, not significant; MTX2, metaxin 2; PKM2, pyruvate kinase M2; H/R, hypoxia/reoxygenation; I/R, ischemia/reperfusion; cKO, cardiac knockout; KO, knockout.

To delineate the molecular domains instrumental in MTX2 and PKM2 interaction, we utilized H-DOCK for molecular docking simulations of complexes MTX2 and PKM2. The interactive residues were predicted based on optimal free energy calculations and structure-based analysis of protein interaction interfaces. The docking results showed a favorable interaction between MTX2 and PKM2, with a calculated free energy of -14.14 kcal/mol (free energy < 0 was considered significant), indicating a positive interaction between MTX2 and PKM2. The predicted 3-dimensional crystal structures of the MTX2-PKM2 complex suggested the potential involvement of the MTX2 TOM37 domain or TRADD domain in its interaction with PKM2 (Figure 5A). Therefore, Flag-tagged MTX2 mutants based on its structural domain were constructed (Flag-MTX2 (Δ41-161 AA) plasmid, Flag-MTX2 (Δ154-195 AA) plasmid) (Figure 5B). *Mtx2* knockout H9C2 cells were generated using the CRISPR-Cas9 technique. We performed immunoprecipitation experiments in *Mtx2* KO H9C2 cells co-expressing plasmids of Flag-MTX2 mutants and GST-PKM. As shown in Figure 5C, PKM2 predominantly precipitated with Flag-MTX2 (full-length MTX2) and Flag-MTX2 (Δ154-195 AA) mutant. However, Flag-MTX2 (Δ41-161 AA) mutant transfection failed to capture the co-expressed GST-PKM2 in *Mtx2* KO H9C2 cells, indicating that MTX2 might primarily interact with PKM2 via its TOM37 domain (41-161 AA). To elucidate whether MTX2 regulates PKM2 tetramerization via interaction with its TOM37 domain (41-161 AA), we overexpressed MTX2 mutants in *Mtx2* KO H9C2 cells after H/R insult. Following glutaraldehyde cross-linking, the proportion of the tetramer in Flag-MTX2 (Δ154-195 AA) mutant overexpressing cells was notably increased, and the dimeric PKM2 reduced when compared with control cells (Figure 5D-E). However, deletion of the 41-161 AA domain in MTX2 abrogated its ability to promote PKM2 tetramerization (Figure 5D-E). To understand whether or not this sequence of MTX2 was associated with its regulation on cardiomyocyte glucose metabolism, we next determined the glycolytic and mitochondrial phenotypes in *Mtx2*-deficient cardiomyocytes overexpressing Flag-MTX2 (full-length MTX2) or Flag-MTX2 (Δ41-161 AA) mutant. Specifically, cKO mice were transfected with adenoviruses to overexpress a control vector, MTX2, or MTX2 (Δ41-161 AA). One week after adenovirus intra-myocardial injection, mice were subjected to I/R surgery. After 3-h reperfusion, cardiomyocytes were isolated for the OCR and ECAR determination. In line with the cross-linking studies, we found that overexpression of MTX2 resulted in a notable elevation in both oxidative phosphorylation and glycolysis when compared to the vector control, whereas MTX2 (Δ41-161 AA) overexpression failed to induce an obvious alteration in either ECAR or OCR (Figure 5F-I). Together, these results suggest that MTX2 orchestrates PKM2 tetramerization through its TOM37 domain (41-161 AA), thereby promoting glucose metabolism in cardiomyocytes.

### Activating PKM2 rescues the exacerbated I/R injury in cKO hearts

**Figure 6:**
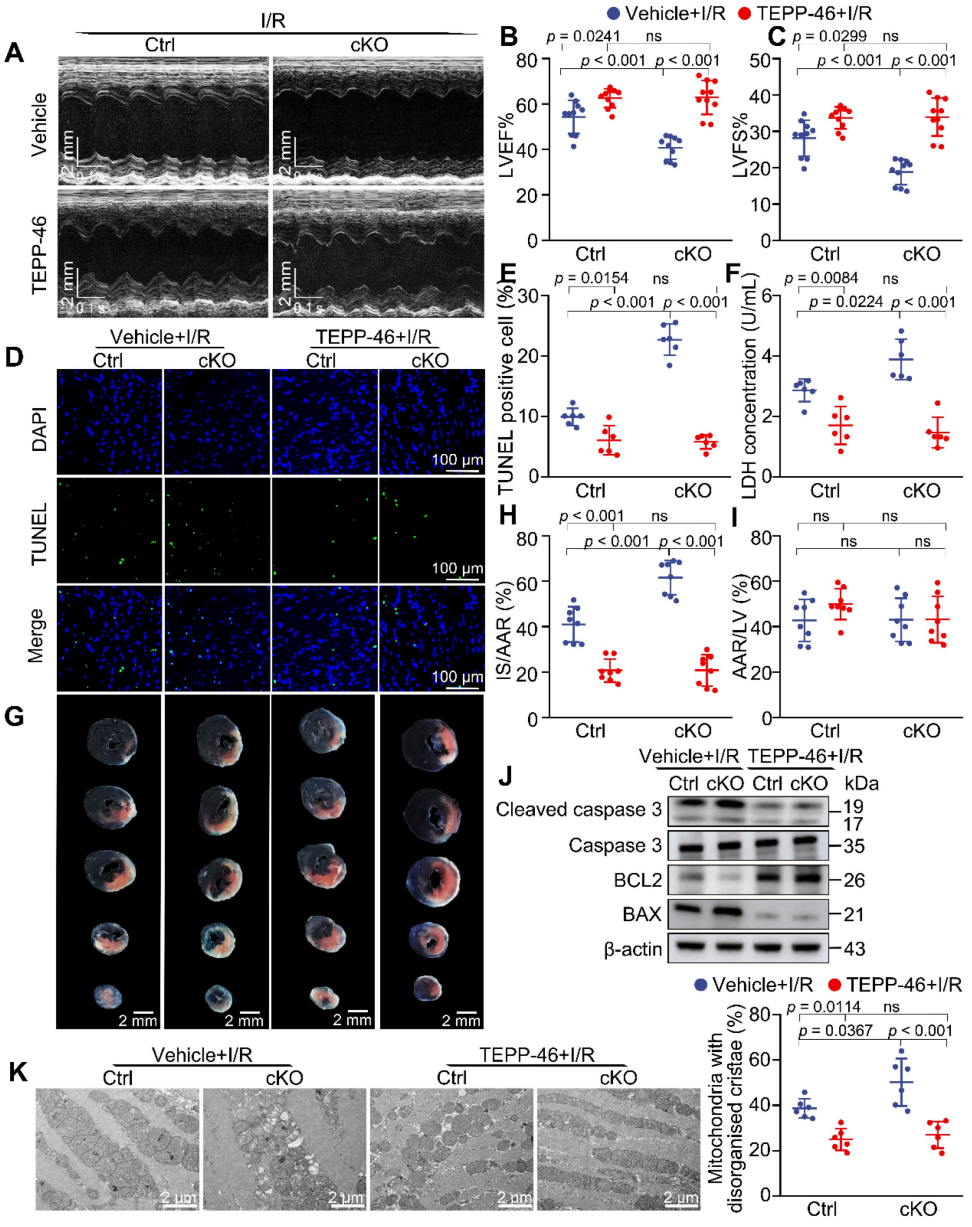
** Activating PKM2 rescues the exacerbated I/R injury in cKO hearts.** 10-week Ctrl mice or cKO mice were intraperitoneally injected with TEPP-46 15 min before sham or I/R surgery. **(A-C)** Representative M-Mode echocardiographic images from and measurements of LV ejection fraction and LV fractional shortening at 24 h after reperfusion in the Ctrl and cKO mice treated with vehicle or TEPP-46 (n = 10). **(D-E)** Representative cardiac apoptosis detected by TUNEL staining after 3-h reperfusion in the Ctrl and cKO mice treated with vehicle or TEPP-46 and TUNEL-positive cardiomyocyte quantification. Green: TUNEL-positive apoptotic nuclei; Blue: DAPI-stained total nuclei. (n = 6). Scale bar: 100 μm. **(F)** Cardiac damage by LDH release assay after 3 h reperfusion in the Ctrl and cKO mice treated with vehicle or TEPP-46 (n = 6). **(G-I)** Representative heart slices stained by Evans blue and tetrazolium chloride (TTC) after 24-h reperfusion in the Ctrl and cKO mice treated with vehicle or TEPP-46. The infarcted size (IS, white), areas of the left ventricle (LV, red plus white plus blue), and areas at risk (AAR, red plus white) were calculated (n = 8). Scale bar: 2 mm. **(J)** Western blot of cardiac cleaved caspase 3, total caspase 3, BAX and BCL2 after 3-h reperfusion in the Ctrl and cKO mice treated with vehicle or TEPP-46 (n = 6). **(K)** Representative transmission electron microscopy (TEM) images and quantitative analysis of the proportion of mitochondria with disorganized cristae in the Ctrl and cKO mice treated with vehicle or TEPP-46 (n = 6). Data were analyzed by two-way ANOVA, followed by a Tukey post-hoc test. All values are presented as mean ± SD. ns, not significant; MTX2, metaxin 2; I/R, ischemia/reperfusion; cKO, cardiac knockout; Ctrl, control.

Given our findings that enhancing PKM2 activity could alleviate the metabolic defect caused by *Mtx2* deficiency, we sought to examine whether reduced PKM2 activity is responsible for exacerbated I/R phenotype in cKO mice. TEPP-46 was intraperitoneally injected 15 min before I/R surgery. Both cKO and their Cre littermates were then subjected to 45-min ischemia and 24-h reperfusion for echocardiographic detection. The cardiac function of the TEPP-46-treated group was significantly improved in comparison with the untreated cKO group, as evidenced by a marked increase in LVEF and LVFS, along with a significant reduction in LVEDD and LVESD (Figure 6A-C, Table S2). Apoptosis induced by *Mtx2* deficiency during I/R was decreased by TEPP-46 treatment, as indicated by decreased TUNEL-positive myocytes, cleaved caspase 3/ caspase 3 ratio and BAX/BCL2 ratio (Figure 6D, 6E, 6J and Figure S10). Furthermore, in response to I/R attack, MTX2 loss-of-function exacerbated LDH release, which was reversed by pharmacological PKM2 activation (Figure 6F). In agreement with these findings, TTC/ Evans blue staining showed that TEPP-46 treated mice exhibited a smaller infarct size compared with cKO mice (Figure 6G-I). Additionally, TEM analysis exhibited an increased proportion of mitochondria with disorganized cristae in cKO heart under I/R attack, which was significantly alleviated by TEPP-46 treatment (Figure 6K).

Importantly, the cKO group recovered to the same level as the Ctrl mice after TEPP-46 administration in response to I/R attack, suggesting that reversing the disturbance of glucose metabolism is sufficient to rescue the pathological mechanisms caused by *Mtx2* deficiency. Together, these findings demonstrate that activating PKM2, which is suppressed by MTX2 loss-of-function, can rescue myocardial apoptosis, infarction, and ventricular dysfunction during I/R.

### Reconstitution of MTX2 in the heart alleviates I/R injury

Since MTX2 was downregulated in the I/R hearts, we proceeded to investigate whether MTX2 reconstitution could rescue the metabolic dysfunction under I/R stress. We utilized an adenovirus carrying *Mtx2* gene to overexpress MTX2 specifically in cardiac tissue. This was achieved through intra-myocardial injection, as depicted in Figure S11A-B. A shift in the distribution of PKM2 from dimers to tetramers was observed in MTX2 overexpressed hearts (Figure S11C-D). Furthermore, by utilizing a seahorse flux analyzer, we found that MTX2 reconstitution improved I/R-induced decline of mitochondrial respiration and glycolytic flux in cardiomyocytes, and the baseline OCR and ECAR were comparable between the mice treated with Ad-EV and Ad-*Mtx2* (Figure S11E-H).

**Figure 7:**
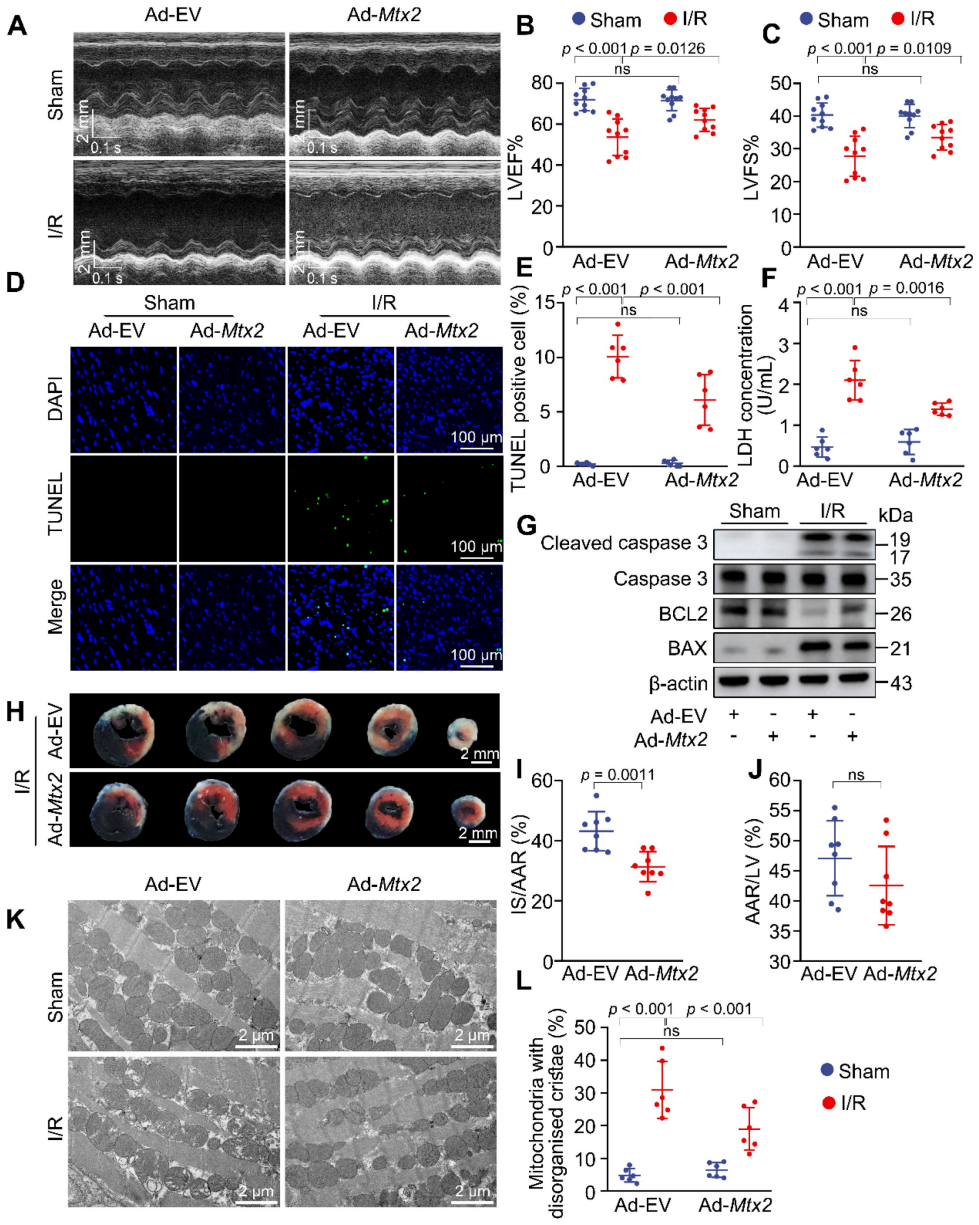
** Reconstitution of MTX2 in the heart alleviates I/R injury.** 10-week mice received intra-myocardial injection with EV or *Mtx2* adenovirus 7 d before sham or I/R operation. **(A-C)** Representative M-Mode echocardiographic images and measurements of LV ejection fraction and LV fractional shortening at 24 h after reperfusion in Ctrl and MTX2-overexpressed mice (n = 10). **(D-E)** Representative cardiac apoptosis detected by TUNEL staining after 3-h reperfusion in Ctrl and MTX2-overexpressed mice and TUNEL-positive cardiomyocyte quantification. Green: TUNEL-positive apoptotic nuclei; Blue: DAPI-stained total nuclei. (n = 6). Scale bar: 100 μm. **(F)** Cardiac damage by LDH release assay after 3-h reperfusion (n = 6). **(G)** Western blot of cardiac cleaved caspase 3, total caspase 3, BAX and BCL2 after 3-h reperfusion in Ctrl and MTX2-overexpressed mice (n = 6). **(H-J)** Representative heart slices stained by Evans blue and tetrazolium chloride (TTC) after 24-h reperfusion in Ctrl and MTX2-overexpressed mice. The infarcted size (IS, white), areas of the left ventricle (LV, red plus white plus blue), and areas at risk (AAR, red plus white) were calculated (n = 8). Scale bar: 2 mm. **(K-L)** Representative transmission electron microscopy (TEM) images and quantitative analysis of the proportion of mitochondria with disorganized cristae after 3-h reperfusion in Ctrl and MTX2-overexpressed mice (n = 6). **(B, C, E, F, L)** Data were analyzed by two-way ANOVA, followed by a Tukey post-hoc test. **(I, J)** Data were analyzed by Two-tailed unpaired Student t-test. All values are presented as mean ± SD. ns, not significant; MTX2, metaxin 2; I/R, ischemia/reperfusion; cKO, cardiac knockout; Ctrl, control.

Given the pivotal role of MTX2 in cardiac PKM2 tetramerization and glucose metabolism, we next sought to determine whether overexpressing MTX2 could protect the heart against cardiac I/R injury. As shown in Figure 7A-C, MTX2 overexpression in the heart significantly alleviated cardiac dysfunction induced by I/R attack as indicated by elevated LVEF and LVFS. Both LVEDD and LVESD were significantly increased in response to I/R attack and Ad-*Mtx2* administration before I/R attack restored the LVEDD to 3.93 ± 0.23 and LVESD to 2.61 ± 0.24 mm (Table S3). Moreover, MTX2 gain-of-function significantly reduced caspase 3 activation, BAX/BCL2 ratio and TUNEL-positive cells in I/R mice, suggesting the I/R-induced apoptosis was mitigated by overexpression of MTX2 (Figure 7D, 7E, 7G and Figure S12). Consistently, the MTX2-overexpressed hearts showed a reduced LDH release, which also confirmed a better recovery compared with the control group in response to I/R attack (Figure 7F). In addition, MTX2 upregulation led to a decrease in cardiac infarct size (Figure 7H-J). As shown in Figure 7K-L, MTX2 overexpression alleviated I/R-induced mitochondria cristae disorder and the appearance of vacuoles. Taken together, these data demonstrate that restoration of MTX2 is capable of ameliorating the detrimental effects of I/R injury.

## Discussion

Myocardial reperfusion leads to compromised metabolism and dysfunctional cardiac performance 26,27. Unfortunately, the underlying mechanism of this damage is far from clear. In this investigation, we report that loss of MTX2 contributes to the disruption of glucose metabolism in the heart in response to I/R stress. We have found that MTX2 interacts with PKM2 through its TOM37 domain to promote the tetramerization of PKM2, thus increasing PK activity. Both increasing MTX2 abundance and PKM2 agonist significantly rescued the reperfusion injury phenotypes. Our results, therefore, reveal for the first time a crucial role of MTX2 in regulating cardiac metabolism and I/R injury.

Metaxin proteins have been implicated in the integration of β-barrel proteins into the OMM 11,28. Very recently, Cartron et al. showed a mechanistic role for MTX in TNF-induced cell death 16,17,29,30. However, whether MTX2, a cytosolic peripherally-associated OMM protein of the MTX family with limited known functions, plays a role in I/R injury remained elusive. Here we have reported, for the first time, a significant decrease in MTX2 expression in the hearts of I/R mice, highlighting its pivotal role as an endogenous regulator of cardiac energy metabolism in response to I/R insult. Cardiac-specific knockdown of *Mtx2* led to impaired mitochondrial OXPHOS and glycolysis in response to I/R. Conditional deletion of *Mtx2* causes a deterioration in classic phenotypes of I/R injury, including impaired cardiac function, enlarged infarct size, increased cardiomyocyte apoptosis, and irregular mitochondrial cristae. To this end, we performed an MTX2 conditional overexpression by intra-myocardial injection adenovirus carrying *Mtx2* and demonstrated a promising therapeutic effect on I/R injury.

Unlike MTX1, MTX2 lacks a mitochondrial C-terminal membrane anchor domain, and is peripherally associated with the outer mitochondrial membrane facing the cytoplasm 15,31, implicating other possible interactions with cytosolic proteins. Our results showed that MTX2 interacts with PKM2 through its TOM37 domain, and is required for the tetramerization of PKM2 in I/R mice. These observations collectively highlight a new role of MTX2 besides its conventional transport function.

The deprivation of oxygen and energy supply induces various myocardial damage during I/R 32,33. Therefore, restoring ATP produced from the glucose metabolism may fulfill the energy demand of cardiomyocytes and is protective against I/R injury. Consistent with this notion, Li et al. found that interventions targeting the PDH flux at reperfusion could increase glucose oxidation and improve the recovery of myocardial function 26. Furthermore, the ischemic pre-conditioning protective mechanism is strongly associated with activated aerobic glycolysis during early reperfusion 34,35. In our study, results from the Seahorse flux analyzer support our hypothesis that MTX2 is instrumental in maintaining the mitochondrial OXPHOS and glycolytic flux during I/R, and PKM2 is identified for its regulation of glucose metabolism. These observations bolster the hypothesis that increased metabolic flux through glycolysis by activating PKM2 would mitigate the accumulation of toxic glucose-derived end products, augment flux through the TCA cycle, and improve mitochondrial function in cardiomyocytes.

As a rate-limiting enzyme for converting phosphoenolpyruvate to pyruvate, PKM2 occupies a crucial regulatory position in glycolytic flux 36,37. Due to its low expression in the normal adult hearts, its function in cardiomyocytes has thus far remained largely unexplored. In this context, it is interesting that *Mtx2* deficiency does not cause a significant change in PKM2 expression during I/R. Instead, the activity of PKM2 is decreased after *Mtx2* knockdown in cardiomyocytes. The mechanism could be a shift in the distribution of PKM2 from tetramers to dimers, with the latter being less catalytically active. Interaction between MTX2 with PKM2 through its TOM37 domain prompts the formation of a tetrameric PKM2, thereby serving as a metabolic switch to augment glycolytic flux in cardiac tissue. Thus, our data revealed that PKM2 functions indispensably for the metabolic control of MTX2 in the I/R hearts.

However, the precise mechanism by which MTX2 regulates this conformational change in PKM2 remains elusive. Liang et.al propose that PKM2 phosphorylation may regulate this interesting transition 38, it is still unknown whether MTX2 induces PKM2 phosphorylation, thereby promoting increased tetramerization. To this end, we conducted additional experiments to investigate the underlying mechanisms. We isolated NRVMs, overexpressed MTX2, subjected them to hypoxia/reoxygenation (H/R) treatment, performed immunoprecipitation (IP) with PKM2 antibody, and immunoblotting (IB) with pan-phosphorylation antibody. The results demonstrated that MTX2 overexpression significantly increased PKM2 phosphorylation levels (Figure S13). Integrating this observation with our present study, we propose that MTX2 might facilitate the conversion of PKM2 dimers to tetramers by enhancing PKM2 phosphorylation. Nonetheless, the detailed mechanisms still await further investigation.

The concept of metabolic therapies has garnered great attention in the management of cardiovascular disease in recent years 39,40. TEPP-46, a small molecule capable of regulating PKM2 polymerization, was reported to be widely implicated in various pathological conditions such as kidney diseases, immune system diseases, and cancer 24,41,42. In our intervention studies, the activation of PKM2 by TEPP-46 improves the pathological glucose metabolism in cKO mice undergoing I/R surgery. Furthermore, our data demonstrated that TEPP-46 confers cardioprotective effects against cKO-exacerbated I/R injury, as evidenced by less cardiomyocyte apoptosis, reduced infarct size, and improved cardiac function. These findings provided a promising candidate for pharmacologically regulating PKM2 allostery in the treatment of cardiac diseases characterized by impaired glucose metabolism and decreased MTX2 expression.

In summary, the present study for the first time provides compelling evidence that MTX2 is an important regulator of maintaining metabolic homeostasis in the I/R hearts through modulating PKM2 tetramer formation. The direct interaction of MTX2 and PKM2 through its TOM37 domain provides new molecular insights into the regulation of PK activity. Considering that MTX2 is downregulated in response to I/R insult, our findings suggest that early intervention targeting MTX2 and PKM2 might be an effective strategy to protect against I/R injury by preserving metabolic homeostasis in the hearts.

## Supplementary Material

Supplementary methods, figures and tables.

## Abbreviations

cKO: conditional knockout
ECAR: extracellular acidification rate
H/R: hypoxia/reoxygenation
I/R: ischemia-reperfusion
LVEF: left ventricular ejection fraction
LVFS: left ventricular fractional shortening
MTX2: metaxin 2
NRVMs: neonatal rat ventricular cardiomyocytes
OCR: oxygen consumption rate
PKM2: pyruvate kinase M2
RNA-seq: RNA sequencing
WT: wild type
