# Supplementary material for: MTX2 facilitates PKM2 tetramerization to promote cardiac glucose metabolism and protects the heart against ischemia/reperfusion injury
Source: Theranostics. 2025 Jun 9;15(14):6737–52. doi: 10.7150/thno.110162 (PMC12203670; doi:10.7150/thno.110162)
Supplement: Supplementary file 1 — Supplementary methods, figures and tables. [file thnov15p6737s1.pdf]

Supplementary Materials

MTX2 facilitates PKM2 tetramerization to promote cardiac glucose metabolism and protects the heart against ischemia/reperfusion injury

Running title: MTX2 Modulates I/R via Glucose Metabolism

Supplemental Methods

Animal studies

Animal studies were performed according to the National Institutes of Health Guidelines for the Care and Use of Laboratory Animals (NIH publication No. 85-23, revised 2011). Animal protocols and performances were approved by the Institutional Animal Care and Use Committee of PLA General Hospital. Euthanasia was performed in accordance with the American Veterinary Medical Association (AVMA) Guidelines for the Euthanasia of Animals (2020) and with the approval of local animal welfare committees.

Adult male C57BL/6J mice were purchased from Model Organisms Center, Inc. (Shanghai, China). Mice were anesthetized by inhalation of 2% isoflurane in oxygen throughout the cardiac I/R surgery, intra-myocardial injection of adenovirus, echocardiography and heart excision for adult cardiomyocyte isolation. Finally, mice were euthanized in their home cage via carbon dioxide inhalation. Carbon dioxide was introduced into the cage at a rate of 3 L/min, added to the existing air in the chamber, until the cessation of respiration in the mice was observed. Mice were then remained in cages for an additional 3 min. Cervical dislocation was performed thereafter to ensure death. The hearts were either stored at −80 °C for subsequent protein extraction or immersed in 4% paraformaldehyde for histological analyses.

Mtx2$^{flox/flox}$ mice and α-MHC-MerCreMer mice (C57BL/6J background) were generated by Model Organisms Center, Inc. (Shanghai, China). The sequences of *Mtx2* cKO mouse genotyping primers were available in Table S4. Briefly, *Mtx2*$^{flox/flox}$ mice were bred with α-MHC-MerCreMer mice to generate cardiac-specific *Mtx2* knockout mice (cKO). Cre recombinase was induced by intraperitoneal administration of tamoxifen (Sigma-Aldrich, T5648) dissolved in 10% ethanol and 90% peanut oil for 5 consecutive days (40 mg/kg of body weight), as previously described [1]. Mice were subjected to I/R surgery after a washout period of 2 weeks.

In this study, in which the *Mtx2* gene was knocked out by Cre recombinase-mediated recombination, corresponding Cre recombinase-expressing strains without the floxed target genes were always utilized as negative controls. The control group was subjected to the same tamoxifen treatment as the experimental group undergoing gene inactivation.

Vehicle or TEPP-46 (50 mg/kg in 40% (w/v) (2-hydroxypropyl)-β-cyclodextrin in water, Sigma-Aldrich, 505487) were administered by intraperitoneal injection 15 min before the onset of myocardial ischemia.

All animals were randomly assigned to different experimental groups. Mice were maintained under identical conditions of temperature (22 ± 0.5 °C), humidity (60 ± 5%), and a 12:12 h light/dark cycle. Standard chow diet and water were available ad libitum.

Myocardial I/R mouse model

The cardiac I/R surgery was performed as previously reported [2]. In brief, male mice were anesthetized with 2% isoflurane and followed by a left lateral thoracotomy to expose the heart. A ligation was performed on the left anterior descending coronary artery (LAD) using a 7-0 silk suture for 45 min to induce ischemia. This was followed by 3 h (for all assays except cardiac function and infarct size) or 24 h (for cardiac function and infarct size) of reperfusion. Sham-operated animals underwent the same procedure except for the LAD occlusion and reperfusion. Mice with unsuccessful surgery (LAD ligation, death prior to the completion of reperfusion etc.) were excluded from the study.

Intra-myocardial injection of adenovirus

Ad-*Mtx2* or Ad-EV adenovirus was designed and synthesized by Hanbio Co., Ltd. (Shanghai, China). The sequences were available in Table S5. Intramyocardial adenovirus injection was performed as previously described [2]. Specifically, the adenovirus was diluted to 2.5×10^{11} PFU/mL. 30 μL of adenovirus was injected into the left ventricular (LV) free wall by using a 30-gauge needle (Hamilton Co. Reno, NV, USA) at 3 sites. Mice were then subjected to I/R or sham surgery 1 week post-adenovirus injection.

Echocardiography

The echocardiographic examination was performed on mice 24 h after reperfusion by a VEVO 2100 echocardiography system (Visual Sonics Inc., Toronto, Canada). Mice were anesthetized by 2% isoflurane inhalation with a heart rate of over 350 beats per minute. Two-dimensional echocardiographic views were captured at the section of the papillary muscle tips below the mitral valve. The left ventricular (LV) dimensions and wall thicknesses were measured. Left ventricular ejection fraction (LVEF) and left ventricular fractional shortening (LVFS) were calculated as previously described [3].

Evaluation of infarct size

Following 24 h of reperfusion, mice were anesthetized by 2% isoflurane and hearts were quickly excised. Mice were intravenously injected with 2% Evans blue dye, frozen in -20 ºC refrigerator for 15 min and sliced into five 1 mm-thick slices. The heart sections were then incubated in 1% triphenyl tetrazolium chloride (TTC, Sigma, T8877) in PBS at 37 ºC for 15 min and digitally photographed, as we previously described [4]. Myocardial infarct size was expressed as a ratio of the infarct area and the area at risk quantified using Image J.

Histological analysis

Immunohistochemistry staining was determined on mouse hearts as we previously described [5]. In brief, 4-mm heart tissues were treated with citric acid antigen retrieval buffer for antigen retrieval and hydrogen peroxide for endogenous peroxidase activity blockage. Subsequently, the sections were incubated with primary MTX2 antibody overnight at 4 °C. Following incubation with the secondary antibody, the sections were visualized with diaminobenzidine (DAB), captured with a Leica microscope, and quantified by Image-Pro Plus software.

Cell isolation and culture

Neonatal rat ventricular cardiomyocytes (NRVMs) were isolated from 2-day-old neonatal Sprague-Dawley rat hearts as previously described [2]. Briefly, the left ventricles

were dissected and digested with 0.1% collagenase type II at 37 °C. The suspending cardiomyocytes were then collected and seeded in Dulbecco's modified Eagle's medium (DMEM). After 2 h differential centrifugation, the non-adherent cells (NRVMs) were collected, plated in dishes, and cultured in the complete medium containing 0.1 mM 5- BrdU (Invitrogen, B23151).

For adult cardiomyocytes isolation, 10-week-old mice were anesthetized with 2% isoflurane and heparinized with heparin. Hearts were excised and perfused in the Langendorff system with perfusion solution (126 mM NaCl, 4.4 mM KCl, 18 mM $NaHCO_3$, 1 mM $MgCl_2$, 11 mM glucose, 10 mM 2,3-butanedione monoxime, 30 mM taurine and 4 mM HEPES). Afterward, enzymatic digestion was initiated by adding collagenase solution (perfusion solution with 0.1% bovine serum albumin, 0.025 mM $CaCl_2$, and 0.1% type II collagenase). After digestion, the myocytes were triturated with a 10 ml pipette at a slow speed, sieved through a 100 μm filter, and exposed to incremental concentrations of Ca^{2+} (range from 0.05 to 0.525 mM). Obtained cardiomyocytes were planted in the laminin-precoated dishes, as previously described [2].

In vitro hypoxia/reoxygenation (H/R) model

For the in vitro cardiac I/R study, cardiomyocytes were subjected to 9-h hypoxia followed by 3-h reoxygenation. To induce hypoxia, cardiomyocytes were placed into a hypoxia chamber (Billups-Rothernberg, US) containing 95% N_2 and 5% CO_2 at 37°C. The reoxygenation process was carried out in a standard incubator with the routine culture medium.

Seahorse assay

An XF24 Extracellular Flux Analyzer (Agilent Seahorse Bioscience, MA, USA) was used to assess the cardiomyocyte metabolic flux. In brief, adult cardiomyocytes were isolated and seeded onto laminin-coated Agilent Seahorse XF24 Cell Culture Microplate (Agilent, V7-PS, 100777) at a density of 1.0×10^4 cells per well. For glucose oxidation detection, the

medium was replaced with XF basal medium supplemented with 5.5 mM glucose, 1 mM sodium pyruvate, and 2 mM GlutaMAX one hour before the assay. For glycolysis function detection, the plating medium was replaced with an XF basal medium supplemented with 4 mM L-glutamine one hour before the assay. For FAO detection, the complete medium was replaced with a substrate-limited medium (DMEM, 0.5 mM glucose, 1 mM GlutaMAX, 0.5 mM carnitine, 1%FBS) overnight. Oxygen consumption rate (OCR) and extracellular acidification rate (ECAR) were measured to evaluate fatty acid oxidation (FAO), glucose oxidation and glycolysis in real-time when the appropriate substrates were injected into the wells at the final concentrations given: Oligomycin (1 μM)、FCCP (0.5 μM) and AA (1.5 μM) for glucose oxidation determination; glucose (10 mM), oligomycin (1 μM), and 2-deoxyglucose (2-DG) (50 mM) for glycolysis determination. oligomycin (2 μM), FCCP (2 μM), and antimycin A (4 μM) for FAO determination. The baseline and stepwise injection measurements were calculated following the XF24 protocol.

Co-immunoprecipitation (co-IP) assay

Extraction of whole-cell lysates proteins used for immunoprecipitation (IP) assay was performed as previously described [1]. The supernatant was immunocaptured with 1 μg anti-Flag/GST antibodies (Flag: Sigma, F1804; GST: Proteintech, HRP-66001) at 4 °C overnight. The subsequent co-IP was determined by using a Pierce Co-immunoprecipitation Kit (Thermo Fisher, 26149) according to the manufacturer's instructions. After the final elution, the samples containing the reducing agent (dithiothreitol) were heated and further used for Western blot analysis.

RNA-sequencing assay

After 45-min ischemia and 3-h reperfusion, total RNA was extracted from heart tissues using RNAisoPLUS (Takara, 9109). The RNA sequencing was performed by Applied Protein Technology Co., Ltd (Shanghai, China).

For transcriptome sequencing library preparation, 1 μg RNA from each sample was used

as input material for the RNA sample preparations with ABclonal mRNA-seq Lib Prep Kit (ABclonal, China). The mRNA purification from total RNA was accomplished using poly-T oligo–attached magnetic beads. Fragmentation was performed by divalent cations under elevated temperature in an Illumina proprietary fragmentation buffer. First-strand cDNA was synthesized using a random hexamer primer, followed by the second-strand cDNA synthesis using DNA Polymerase I and RNase H. After repair and A tailing, the double-strand cDNA products were ligated with an adapter, and subjected to PCR amplification through a 14-cycle procedure. Products were purified using the AMPure XP system and library quality was carried out using Agilent Bioanalyzer 4150 system. The library was then sequenced on an MGISEQ-T7 instrument. Clean Reads were aligned using HISAT2 and the fragments per kilobase of transcript sequence per million (FPKM) data were used for further analysis. Differential expression analysis of the gene expression matrix was performed using the DESeq2 package ($|\text{fold change}| \geq 1.0$, $P < 0.05$). Gene Ontology (GO) enrichment analysis of differentially expressed genes was performed and the results were visualized with the OmicShare tool (https://www.omicshare.com/tools/Home/Soft/enrich_circle). GO terms with corrected P value < 0.05 were considered statistically significant. Gene set enrichment analysis (GSEA) was performed with GSEA 4.1.0 software. The strength of the enrichment was ranked by Normalized Enrichment Score (NES) using the Molecular Signature Database (MSigDB) 3.0.

Silver staining and LC-MS/MS analysis for MTX2-interacting proteins

Silver staining was performed using the Fast Silver Stain Kit (Beyotime, P0017S) according to the manufacturer's instructions. Liquid chromatography tandem mass spectrometry (LC-MS/MS) analysis was performed at Applied Protein Technology Co., Ltd (Shanghai, China) using Q Exactive HF-X mass spectrometer (Thermo Fisher Scientific, Waltham, MA, USA) coupled with EASY-nLC 1000 system (Thermo Fisher Scientific, Waltham, MA, USA). In brief, protein samples were electrophoresed on SDS-PAGE gels,

which were subsequently excised and subjected to in-gel tryptic digestion. After dissolved in solvent A (0.1% formic acid, wt/vol.), tryptic peptides were injected into the trap column for separation using loading buffer B (0.1% formic acid in 84% ACN, wt/vol.). Afterward, the samples were injected into the mass spectrometer at a constant column-tip flow rate of 600 nL/min. A full scan range was set from 300 to 1500 with a resolution of 60,000 (at 200 m/z). Spectra data were scrutinized using the Uniprot_rat database (http://www.uniprot.org/) by Proteome Discoverer™ Software 1.4 (Thermo Fisher Scientific, Waltham, MA, USA).

Chemical cross-linking assay

NRVMs were lysed with sodium phosphate buffer (pH = 7.3) containing 0.5% Triton X-100 and 1 × protease inhibitor cocktail (CWbio, CW2200S) for 40 min at 4 °C. The lysates were centrifuged at 12, 000 g for 15 min at 4 °C. The lysates (4 mg/ml) were crosslinked with 0.01% glutaraldehyde for 1 min at 37 °C. The reaction was terminated by the addition of 1 M Tris buffer (pH = 8.0) to reach a final concentration of 50 mM of Tris (pH = 8.0). Samples were then analyzed by Western blot with indicated antibodies.

Glutathione S-transferase (GST) pull-down assay

The GST pull-down assay was performed using Pierce GST Protein Interaction Pull-Down Kit (Thermo Fisher, 21516). Recombinant GST, GST-PKM2, and His-MTX2 proteins were expressed in E. coli strain BL21 and subsequently purified using glutathione-sepharose and Ni-NTA agarose beads according to the manufacturer’s protocol. Control GST and GST-PKM2 recombinant proteins were recombined into sepharose beads at 4 °C for 1 h. The beads were then incubated with His-MTX2 protein at 4 °C for another 1 h. The samples were centrifuged at 1, 000 g for 1 min to obtain the prey fraction. Subsequently, the mixture was dissociated from the beads by elution buffer, washed with incubation buffer, boiled with 4 × SDS-PAGE loading buffer, and subjected to immunoblotting analysis.

Transmission electronic microscopy (TEM)

Heart samples for TEM analysis were prepared as we previously described [1]. Briefly,

LV wall samples were fixed at 4 °C with 2% glutaraldehyde in a 0.1 M sodium cacodylate buffer 3 h after reperfusion. Subsequently, samples were postfixed in 1% osmium tetroxide for 1 h. All images were obtained under a Hitachi H600 Electron Microscope (Hitachi, Japan). In each heart sample, 5 random fields were captured under transmission electron microscope. In each field, at least 20 mitochondria were randomly selected and analyzed to assess the percentage of disorganized cristae.

Measurement of the Pyruvate Kinase Activity

The pyruvate kinase (PK) activity was determined using a pyruvate kinase assay kit (Sigma, MAK072). In brief, heart tissues from different groups were homogenized in the Pyruvate Kinase Assay Buffer and centrifuged at 15,000 × g for 10 minutes to remove the debris. The supernatant was carefully transferred to a 96-well plate, followed by the addition of the Reaction Mix to each well. PK activity was then quantified by measuring the absorbance at 570 nm, in accordance with the manufacturer's instruction.

Generation of *Mtx2* Mutants

To investigate the functional domains of MTX2, we first generated *Mtx2*-knockout (KO) H9C2 cells using CRISPR-Cas9 technology, as we previously described [1]. A single guide RNA (sgRNA; 5′- ACCTTATATCAGCAACTGAG AGG -3′) was subcloned into the lentiCRISPR v2 vector (52,961, Addgene, MA, USA) following hybridization and phosphorylation of oligos. The packaging plasmids pMD2.G (12,259, Addgene, MA, USA), psPAX2 (12,260, Addgene, MA, USA), along with the lentiCRISPRv2-gRNA construct were co-transfected into HEK293T cells for 48 h to generate lentivirus. Afterwards, lentivirus supernatants were collected, and used to infect H9C2 cells in the presence of polybrene (8 μg/ml, C0351, Beyotime, Beijing, China). The H9C2 cells were seeded into 96-well plates to acquire stable monoclonal *Mtx2* knockout H9C2 cell line after puromycin selection to ensure successful integration. To generate different M*tx2* mutants, *Mtx2*-KO H9C2 cells were transfected with mutant plasmids using Lipofectamine 3000. The mutant plasmids were

constructed by Genomeditech (Shanghai, China), with detailed sequences provided in Supplementary Table S6.

Quantitative real-time polymerase chain reaction (RT-PCR)

Total RNA was extracted from frozen heart tissues or cultured cells RNAisoPLUS (Takara, 9109). The cDNAs were prepared using the PrimeScript RT Reagent Kit (Takara, R047A). RT-PCR analysis was performed by SYBR Green Master Mix (Cwbio, CW0957). Sequences of RT-PCR primers were listed as followed: *Pkm2*: Forward: 5′-GTCTGGAGAAACAGCCAAGG-3′; Reverse: 5′-CGGAGTTCCTCGAATAGCTG-3′; *Mtx2*: Forward: 5′-AGATCGCAGCTACAGAACCTTGG-3′; Reverse: 5′-CACACCACTTTGACAGGCAGATTAC-3′.

Western blot analysis

Protein extraction was performed using RIPA buffer with a complete protease inhibitor cocktail and phosphatase inhibitor, as we previously described [6]. For Western blot analysis, protein samples derived from heart tissues or cardiomyocytes were electrophoresed on SDS-PAGE gels and transferred onto nitrocellulose membranes. Afterward, the membranes were blocked in 5% non-fat milk for 1 h and incubated with the following primary antibodies overnight at 4 °C. The primary antibodies were as follows: β-actin (1:1000, Abcam, Ab6276), Caspase 3 (1:1000, Abclonal, A0214), Cleaved caspase-3 (1:1000, Cell Signaling Technology, 9661), FLAG (1:1000, Sigma-Aldrich, F1804), GST(1:15000, Proteintech, HRP-66001), His (1:5000, Proteintech, 66005-1-Ig), MTX2 (1:1000, Abclonal, A7958), PKM1 (1:1000, Cell Signaling Technology, 7067), PKM2 (1:1000, Cell Signaling Technology, 4053).

After washing with PBST 5 times, the membranes were incubated with secondary antibodies, visualized with an ECL Western blot detection kit and scanned by the ChemiDocXRS system (Bio-Rad Laboratory, Hercules, CA, USA).

Statistical analysis

All values were presented as mean ± SD. Data were analyzed using GraphPad Prism 8

statistic software. The normality of the data was assessed using the Shapiro-Wilk test. For normally distributed data, Student's t-test was carried out to compare differences between two groups, and one-way or two-way ANOVA followed by Tukey post-hoc test was used for multiple group comparisons. For non-normally distributed data, Mann-Whitney U test was used for two-group comparisons, and Kruskal-Wallis test with Dunn multiple comparison test was applied for comparisons involving multiple groups. P values < 0.05 were considered statistically significant.

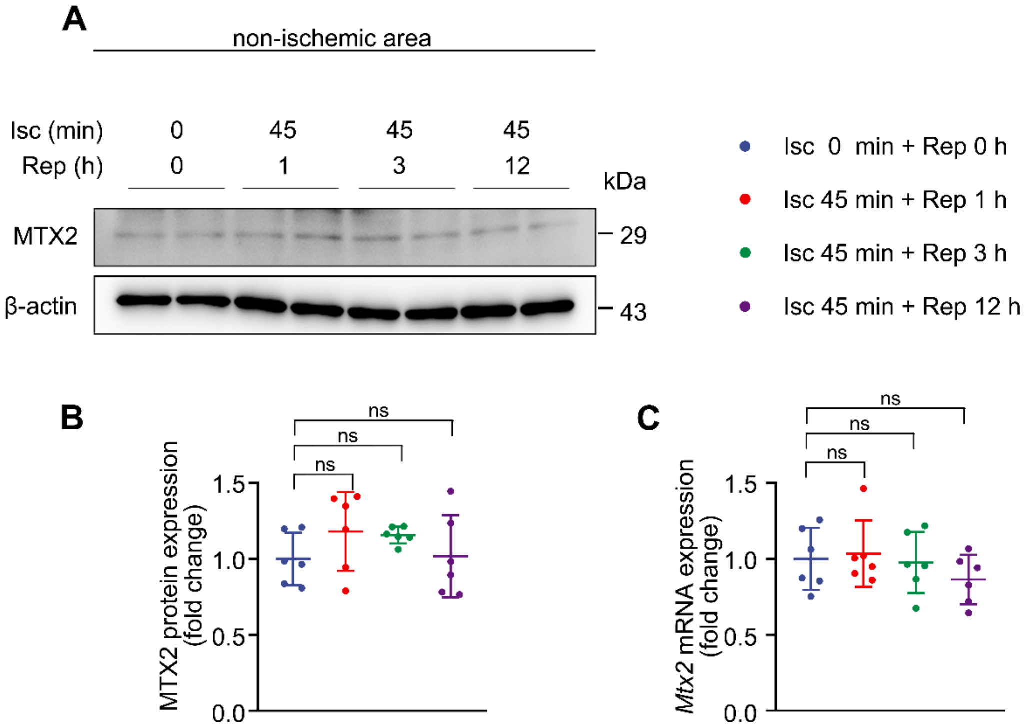

Figure S1. MTX2 expression remains unchanged in non-ischemic area of I/R hearts. 10-week WT mice were subjected to 45-min ischemia followed by reperfusion at different timepoints. (A-B) Western blot and associated quantification of MTX2 protein levels in non-ischemic myocardial tissue at the indicated time points after ischemia/reperfusion (I/R) induction (n = 6). (C) Quantitative real-time polymerase chain reaction (qPCR) analysis of heart *Mtx2* levels in non-ischemic myocardial tissue at the indicated time points after I/R induction (n = 6). (B) Data were analyzed by one-way ANOVA followed by Tukey post-hoc test. (C) Data were analyzed by Kruskal-Wallis test followed by Dunnett's post hoc test. All values are presented as mean ± SD. ns, not significant; MTX2, metaxin 2; Isc, ischemia; Rep, reperfusion; I/R, ischemia/reperfusion; H/R, hypoxia/reoxygenation.

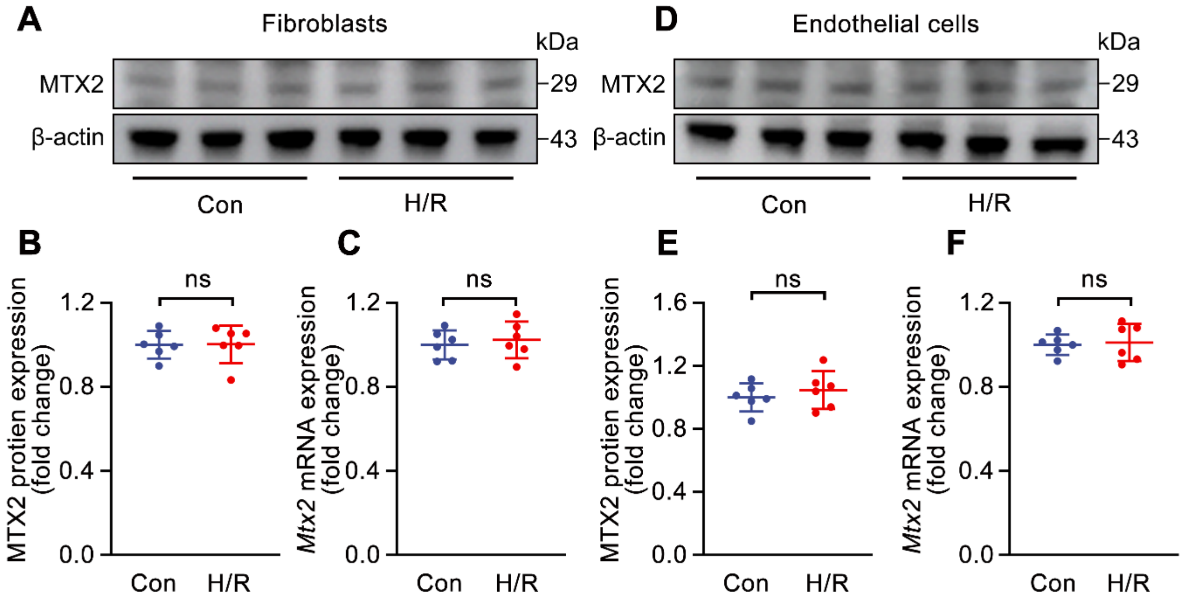

Figure S2. MTX2 expression remains unchanged in neonatal rat cardiac fibroblasts and endothelial cells following H/R exposure. In vitro, neonatal rat cardiac fibroblasts and endothelial cells were isolated and treated with 9-h of hypoxia and 3-h of reoxygenation (H/R) to simulate the ischemia/reperfusion (I/R) injury. (A-B) Western blot and associated quantification of MTX2 protein levels in reoxygenated fibroblasts (n = 6). (C) qPCR analysis of *Mtx2* mRNA levels in fibroblasts under H/R condition (n = 6). (D-E) Western blot and associated quantification of MTX2 protein levels in reoxygenated endothelial cells (n = 6). (F) qPCR analysis of *Mtx2* mRNA levels in endothelial cells under H/R condition (n = 6). (B, C, E, F) Data were analyzed by Two-tailed unpaired Student t-test. All values are presented as mean ± SD. ns, not significant; MTX2, metaxin 2; H/R, hypoxia/reoxygenation.

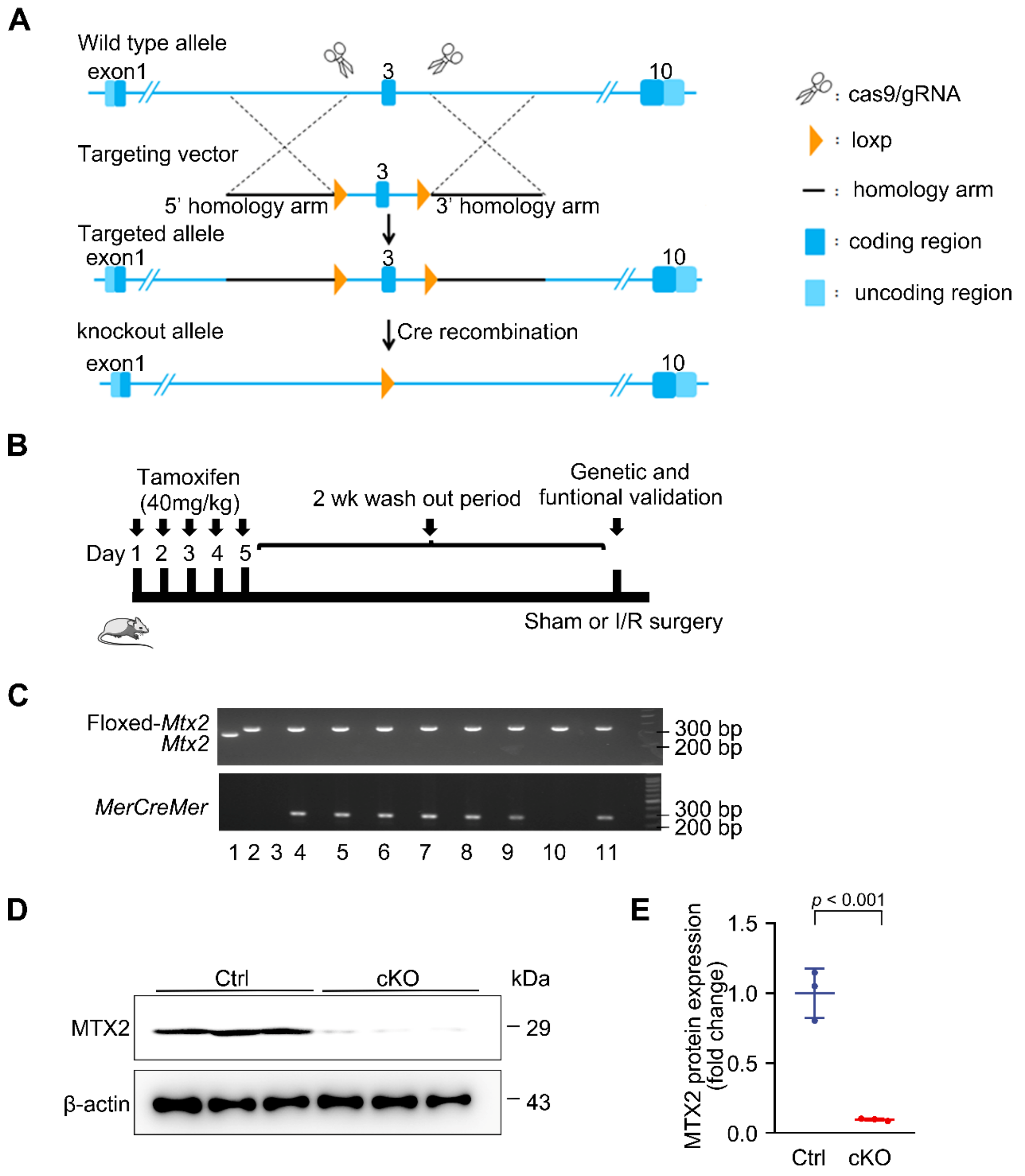

Figure S3. Establishment of cardiac-specific *Mtx2* KO mice. (A) Strategy of generating cardiac-specific *Mtx2* knock-out model. The numbers represent *Mtx2* exons. Triangles indicate the loxP sites. (B) Timeline of tamoxifen-induced *Mtx2* knockout in the adult *MCM*$^{+}$; *Mtx2*$^{f/f}$ mice hearts. (C) Genotyping of *Mtx2* cKO mice (WT: Line1 H_2O: Line 3 cKO: Line 2, 4, 5, 6, 7, 8, 9, 10, 11). (D-E) Western blot and quantification of cardiac MTX2 protein levels in Cre (Ctrl) and *Mtx2* cKO (cKO) mice (n = 3). Data were analyzed by Two-tailed unpaired Student t test. All values are presented as mean ± SD. MTX2, metaxin 2; I/R,

ischemia/reperfusion; cKO, cardiac knockout; Ctrl, control.

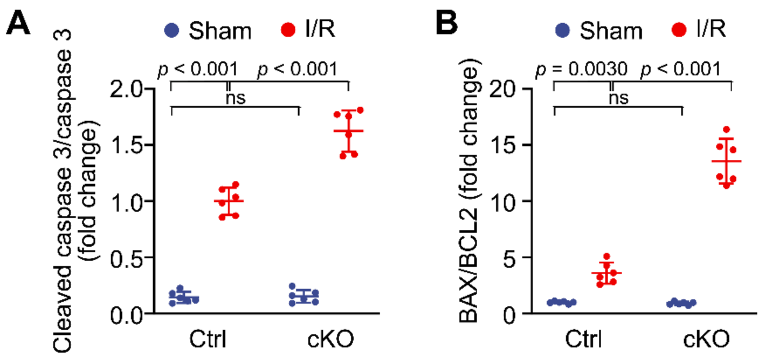

Figure S4. Apoptosis levels in Ctrl and cKO mice. (A) Associated quantification of cardiac cleaved and total caspase-3 after 3 h reperfusion in Ctrl and cKO mice (n = 6). (B) Quantitative analysis of BAX/BCL-2 ratio after 3 h reperfusion in Ctrl and cKO mice (n = 6). (A, B) Data were analyzed by two-way ANOVA, followed by a Tukey post-hoc test. All values are presented as mean ± SD. ns, not significant; I/R, ischemia/reperfusion; cKO, cardiac knockout; Ctrl, control.

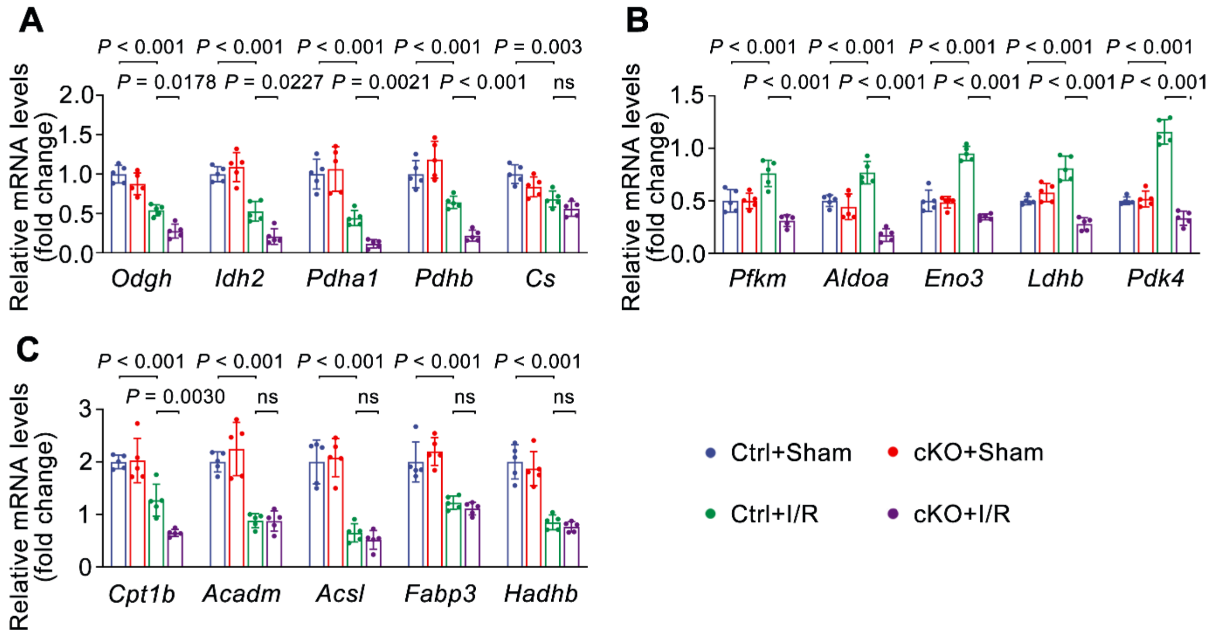

Figure S5. mRNA levels of key metabolic enzymes in Ctrl and cKO mice under I/R attack. 10-week control (Ctrl) mice or *Mtx2* cKO (cKO) mice were subjected to sham or I/R surgery. (A) mRNA levels of *Ogdh*, *Idh2*, *Pdha1*, *Pdhb*, and *Cs* by real-time PCR, normalized to β-actin (n = 5). (B) mRNA levels of *Pfkm*, *Aldoa*, *Eno3*, *Ldhb*, *Pdk4* by real-time PCR, normalized to β-actin (n = 5). (C) mRNA levels of *Cpt1b*, *Acadm*, *Acsl*, *Fabp3*, *Hadhb* by real-time PCR, normalized to β-actin (n = 5). Sequences of primers used were listed in Table S7. Data were analyzed by two-way ANOVA, followed by a Tukey post-hoc test. All values are presented as mean ± SD. ns, not significant; MTX2, metaxin 2; I/R, ischemia/reperfusion; cKO, cardiac knockout; Ctrl, control.

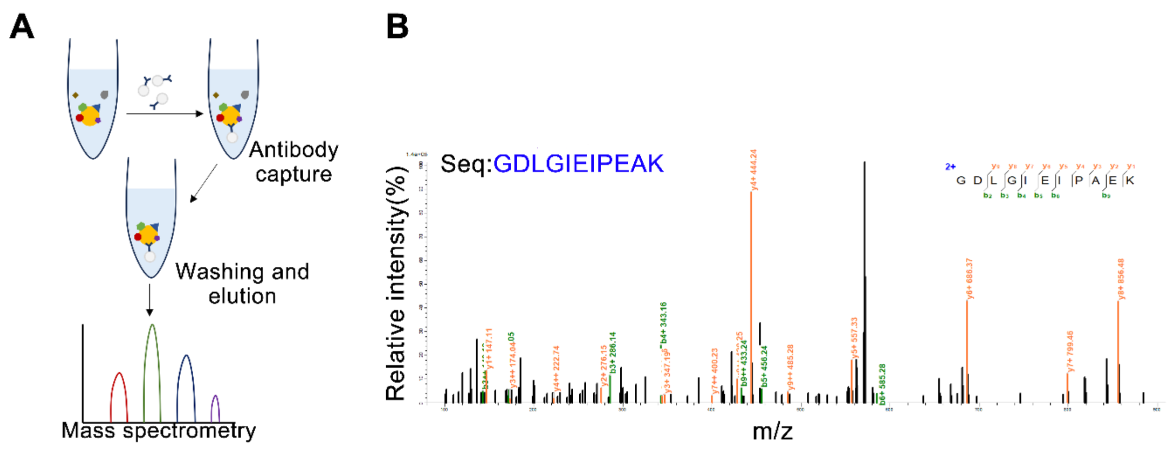

Figure S6. Identification of the peptide PKM (Pyruvate kinase Muscle Isozyme) as an MTX2-interacting target. (A) Illustrations of the experimental approach to screen proteins interacting with MTX2 using co-immunoprecipitation-mass spectrometry analysis. (B) PKM peptide derived from the mass spectrometric analysis.

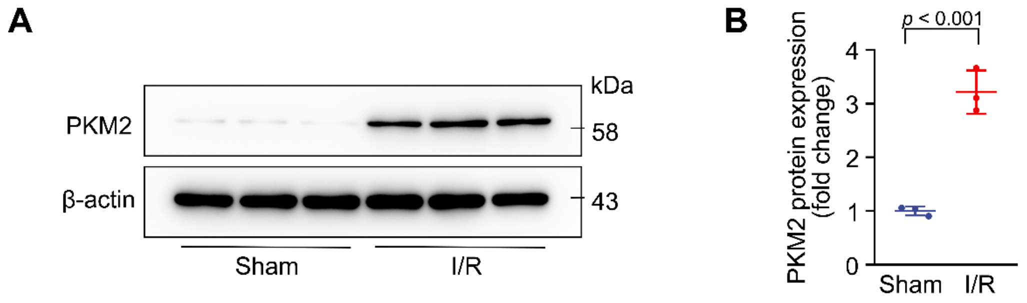

Figure S7. PKM2 is induced in response to I/R attack. (A-B) Representative Western blot images and quantification of cardiac PKM2 protein levels in WT mice (n = 3). Data were analyzed by Two-tailed unpaired Student t test. All values are presented as mean ± SD. PKM2, pyruvate kinase M2; I/R, ischemia/reperfusion.

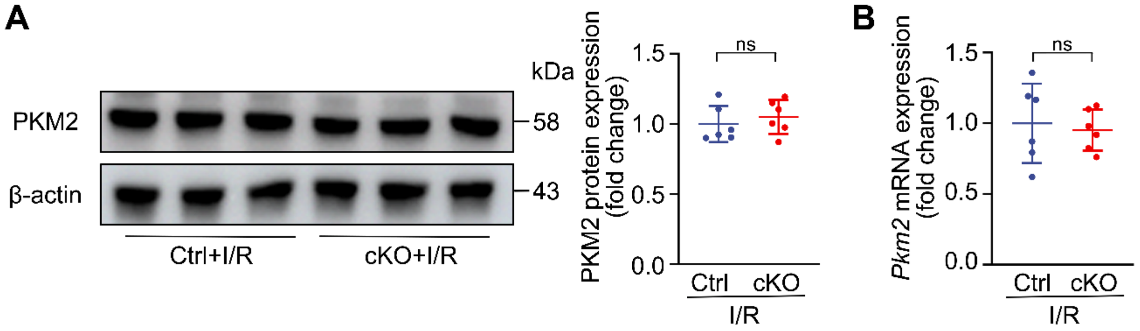

Figure S8. ***Mtx2*** **deficiency does not change PKM2 expression in I/R hearts.** 10-week Ctrl mice or cKO mice were subjected to I/R surgery. (A) Western blot and associated quantification of PKM2 protein levels in Ctrl and cKO mice after 3 h reperfusion (n = 6). (B) qPCR analysis of *Pkm2* mRNA levels in Ctrl and cKO mice under I/R attack (n = 6). Data were analyzed by Two-tailed unpaired Student t test. All values are presented as mean ± SD. ns, not significant; PKM2, pyruvate kinase M2; I/R, ischemia/reperfusion; cKO, cardiac knockout; Ctrl, control.

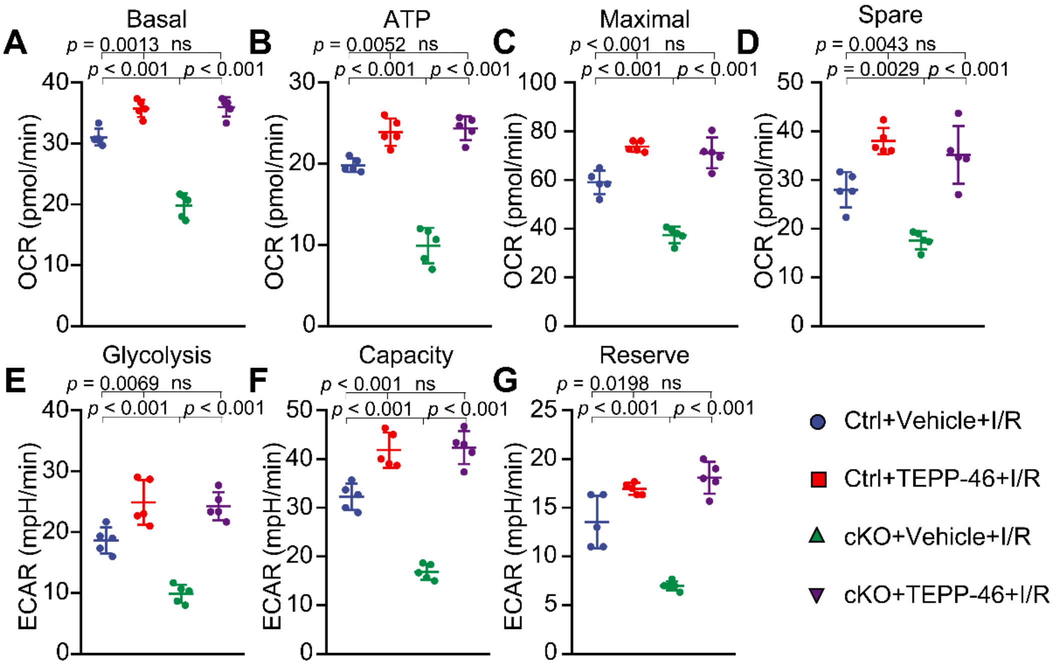

Figure S9. Influence of TRPP-46 on cardiac glucose metabolism in Ctrl and cKO mice. Ctrl or cKO mice were intraperitoneally injected with TEPP-46 15 min before I/R surgery. After 3-h reperfusion, adult cardiomyocytes were isolated for OCR and ECAR determination using a seahorse flux analyzer. (A-D) Quantification of basal respiration, maximal respiration, ATP production, and spare capacity according to instruction (n = 5). (E-G) Quantification of glycolysis, glycolytic capacity, and glycolytic reserve according to instruction (n = 5). Data were analyzed by two-way ANOVA, followed by a Tukey post-hoc test. All values are presented as mean ± SD. ns, not significant; I/R, ischemia/reperfusion; cKO, cardiac knockout.

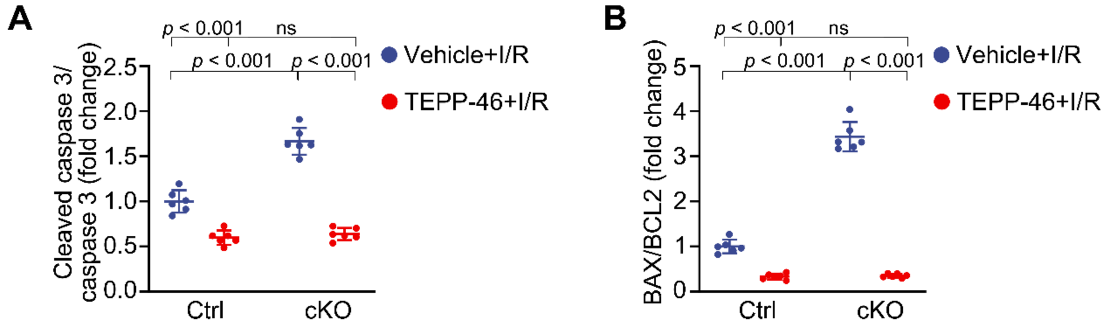

Figure S10. Influence of TRPP-46 on cardiac apoptosis in Ctrl and cKO mice. (A) Associated quantification of cardiac cleaved and total caspase-3 after 3 h reperfusion in the Ctrl and cKO mice treated with vehicle or TEPP-46 (n = 6). (B) Quantitative analysis of BAX/BCL-2 ratio after 3 h reperfusion in Ctrl and cKO mice (n = 6). (A, B) Data were analyzed by two-way ANOVA, followed by a Tukey post-hoc test. All values are presented as mean ± SD. ns, not significant; I/R, ischemia/reperfusion; cKO, cardiac knockout.

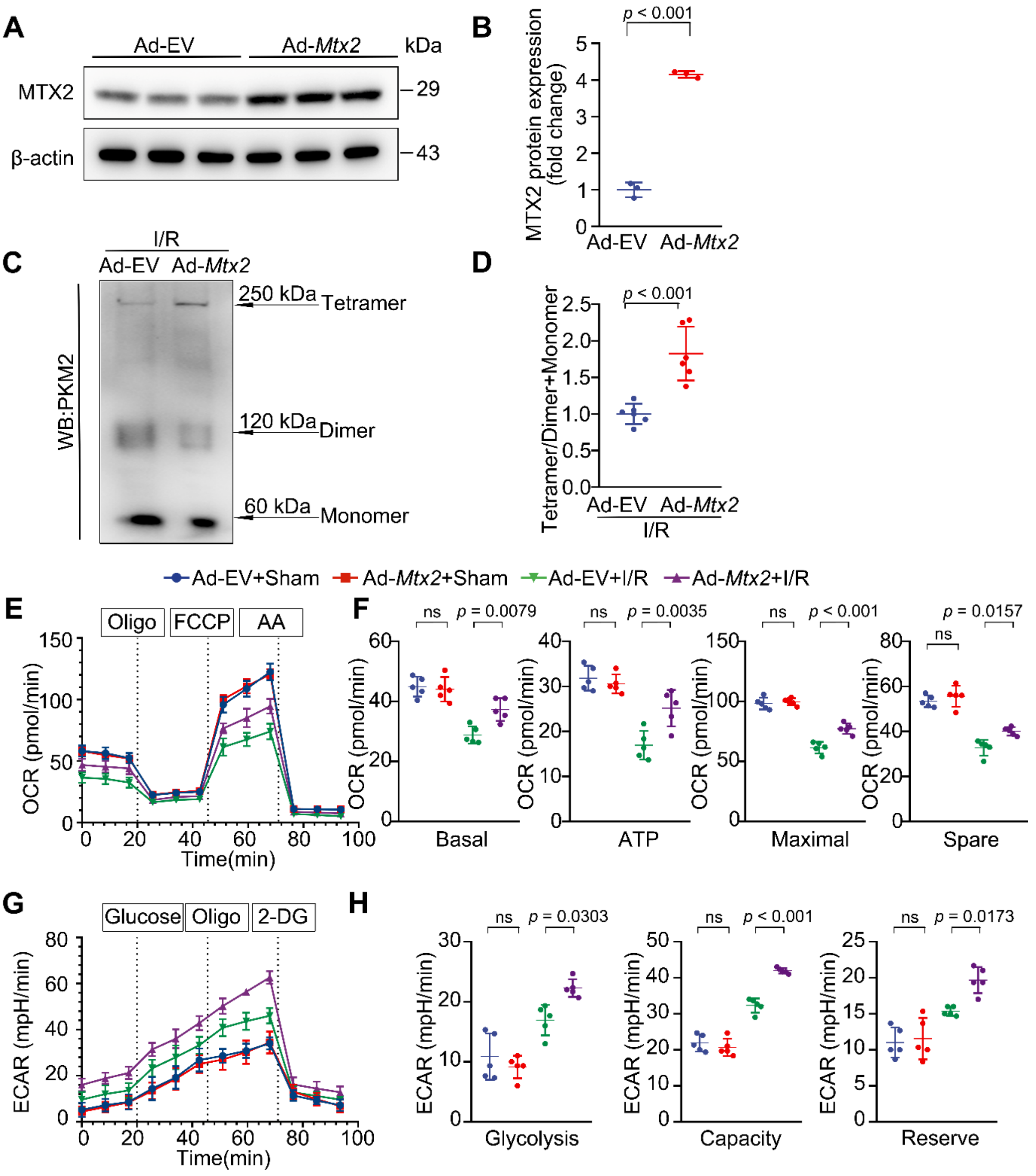

Figure S11. Reconstitution of MTX2 rescues metabolic defect in I/R hearts. (A-B) Representative Western blot images demonstrating MTX2 overexpression efficiency, with associated quantification on the right (n = 3). (C-D) Representative Western blot image and associated quantification of cardiac PKM2 oligomerization after 3 h reperfusion in control and MTX2-overexpressed mice (n = 6). (E-F) OCR curve of adult cardiomyocytes isolated from WT mice with and without overexpression of MTX2 in the presence of I/R attack (n =

5). (F) Quantification of basal respiration, maximal respiration, ATP production and spare capacity according to instruction (n = 5). (G) ECAR curve of adult cardiomyocytes isolated from WT mice with and without overexpression of MTX2 in the presence of I/R attack (n = 5). (H) Quantification of glycolysis, glycolytic capacity and glycolytic reserve according to instruction (n = 5). (B, D) Data were analyzed by Two-tailed unpaired Student t test. (F, H) Data were analyzed by two-way ANOVA, followed by a Tukey post-hoc test. All values are presented as mean ± SD. ns, not significant; MTX2, metaxin 2; I/R, ischemia/reperfusion.

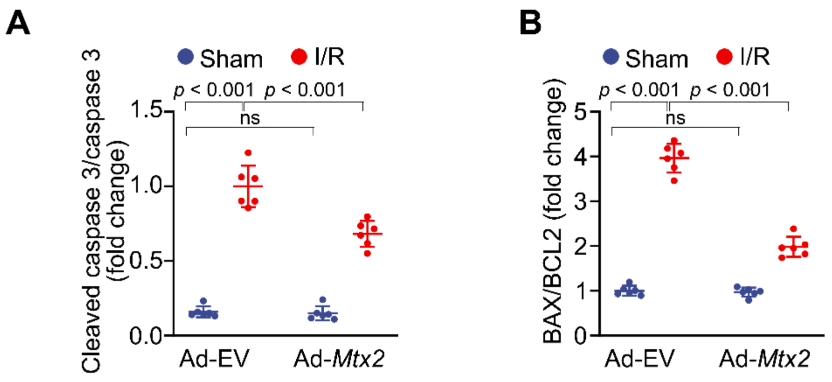

Figure S12. Reconstitution of MTX2 in the heart alleviates I/R-induced apoptosis. (A) Associated quantification of cardiac cleaved and total caspase-3 after 3 h reperfusion in Ctrl and MTX2-overexpressed mice (n = 6). (B) Quantitative analysis of BAX/BCL-2 ratio after 3 h reperfusion in Ctrl and cKO mice (n = 6). (A, B) Data were analyzed by two-way ANOVA, followed by a Tukey post-hoc test. All values are presented as mean ± SD. ns, not significant; MTX2, metaxin 2; I/R, ischemia/reperfusion.

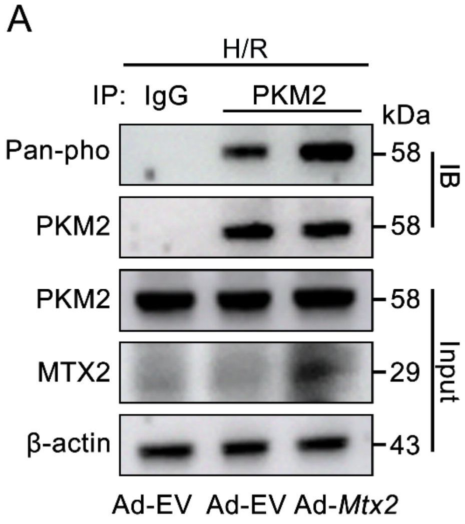

Figure S13. Reconstitution of MTX2 in the NRVMs increases PKM2 phosphorylation under H/R attack. NRVMs were isolated, overexpressed with MTX2, subjected to hypoxia/reoxygenation (H/R) treatment, immunoprecipitated with PKM2 antibody, and immunoblotted with pan-phosphorylation antibody. (A) The phosphorylation level of PKM2 after 3-h of reoxygenation in control and MTX2-overexpressed cardiomyocytes (n = 6). H/R, hypoxia/reoxygenation.

Table S1. Other echocardiographic assessment of cardiac functions in Ctrl mice and cKO mice under I/R attack.

Parameters	Sham		I/R	
	Ctrl	cKO	Ctrl	cKO
**HR, bpm**	492 ± 25	487 ± 21	495 ± 29	486 ± 26
**LVEDD, mm**	3.70 ± 0.34	3.78 ± 0.35	4.27 ± 0.26*	4.71 ± 0.14†
**LVESD, mm**	2.23 ± 0.33	2.32 ± 0.41	3.00 ± 0.45*	3.79 ± 0.26†
**LVEF, %**	71.54 ± 6.17	69.34 ± 7.21	57.41 ± 10.06*	40.83 ± 6.44†
**LVFS, %**	39.76 ± 4.02	38.37 ± 5.06	28.25 ± 7.01*	19.21 ± 3.84†
**IVSWT, mm**	0.93 ± 0.13	0.89 ± 0.17	0.82 ± 0.09	0.77 ± 0.08
**PWT, mm**	0.77 ± 0.09	0.77 ± 0.12	0.79 ± 0.09	0.80 ± 0.08

HR: heart rate, LVEDD: LV end-diastolic diameter, LVESD: LV end-systolic diameter, LVEF: left ventricular ejection fraction, LVFS: left ventricular fractional shortening, IVSWT: interventricular septal wall thickness, PWT: posterior wall thickness. Values are means ± SD. *p < 0.05 vs. Ctrl + Sham group, †p < 0.05 vs. Ctrl + I/R group.

Table S2. Other echocardiographic assessment of cardiac functions in Ctrl mice and cKO mice after TEPP-46 administration in response to I/R attack.

Parameters	Vehicle + I/R		TEPP-46 + I/R	
	Ctrl	cKO	Ctrl	cKO
**HR, bpm**	488 ± 21	492 ± 26	492 ± 28	501 ± 19
**LVEDD, mm**	4.25 ± 0.37	4.68 ± 0.27*	3.72 ± 0.35*	3.78 ± 0.24†
**LVESD, mm**	3.06 ± 0.38	3.81 ± 0.32*	2.47 ± 0.31*	2.50 ± 0.32†
**LVEF, %**	54.88 ± 7.15	39.98 ± 4.25*	62.62 ± 4.12*	62.92 ± 7.65†
**LVFS, %**	27.82 ± 5.12	18.85 ± 3.02*	34.10 ± 2.95*	33.97 ± 5.43†
**IVSWT, mm**	0.82 ± 0.10	0.77 ± 0.14	0.84 ± 0.10	0.86 ± 0.11
**PWT, mm**	0.81 ± 0.10	0.80 ± 0.13	0.80 ± 0.09	0.81 ± 0.12

HR: heart rate, LVEDD: LV end-diastolic diameter, LVESD: LV end-systolic diameter, LVEF: left ventricular ejection fraction, LVFS: left ventricular fractional shortening, IVSWT: interventricular septal wall thickness, PWT: posterior wall thickness. Values are means ± SD. *$p < 0.05$ vs. Ctrl + Vehicle + I/R group, †$p < 0.05$ vs. cKO + Vehicle + I/R group.

Table S3. Other echocardiographic assessment of cardiac functions in mice injected with adenovirus carrying scramble (Ad-EV) or *Mtx2* (Ad-*Mtx2*).

Parameters	Sham		I/R	
	Ad-EV	Ad-*Mtx2*	Ad-EV	Ad-*Mtx2*
**HR, bpm**	487 ± 18	495 ± 24	490 ± 22	499 ± 26
**LVEDD, mm**	3.54 ± 0.40	3.52 ± 0.29	4.37 ± 0.26*	3.93 ± 0.23†
**LVESD, mm**	2.11 ± 0.19	2.11 ± 0.19	3.17 ± 0.36*	2.61 ± 0.24†
**LVEF, %**	72.04 ± 5.13	70.95 ± 5.65	54.02 ± 9.30*	62.19 ± 6.03†
**LVFS, %**	40.31 ± 3.32	39.11 ± 3.74	27.68 ± 6.21*	33.09 ± 4.13†
**IVSWT, mm**	0.89 ± 0.07	0.88 ± 0.10	0.84 ± 0.04	0.86 ± 0.06
**PWT, mm**	0.76 ± 0.10	0.81 ± 0.11	0.77 ± 0.10	0.81 ± 0.12

HR: heart rate, LVEDD: LV end-diastolic diameter, LVESD: LV end-systolic diameter, LVEF: left ventricular ejection fraction, LVFS: left ventricular fractional shortening, IVSWT: interventricular septal wall thickness, PWT: posterior wall thickness. Values are means ± SD. *$p < 0.05$ vs. Ad-EV + Sham group, †$p < 0.05$ vs. Ad-EV + I/R group.

Table S4: Sequences of *Mtx2*-cKO mouse genotyping primers.

Primer	Sequence 5' --> 3'	Primer Type
CKO- Genotyping I	TCCAGGTGTCAGCAACGAAA	Forward
CKO- Genotyping II	ATGCACCGCACACCACTTAT	Reverse
CKO- Genotyping III	TTGTCCACTGACGGTTGAGC	Forward
CKO- Genotyping IV	GTGCACTGGTGCTTTTGCTG	Reverse

Table S5. Sequences of Adenovirus.

Adenovirus		Sequence
Mtx2	Adeasy-m-Mtx2-K/X-F	ctgtgaccggcgcctactctggtaccgccaccATGTCTCTGGTGGCGGAAGC
	Adeasy-m-Mtx2-K/X-R	TGTCATCGTCATCCTTGTAGTCctcgagAGACAACCTGCCTTTACCCC

Table S6: Sequence of Mouse *Mtx2* mutant plasmid

Plasmids	Sequence
**Mouse_*Mtx2* (p.154-195del) CDS**	GCCACCATGTCTCTGGTGGCGGAAGCGTTCGTCTCCCAGA TCGCAGCTACAGAACCTTGGCCTGAAAATGCGACCTTGTA TCAGCAACTGAGAGGGGAGCAAATTTTGCTTTCTGACAAT GCAGCATCTCTTGCTGTGCAGGCATTTTTGCAGATGTGTAA TCTGCCTGTCAAAGTGGTGTGTAGGGCAAATGCGGAATAT ATGTCTCCATCTGGAAAAGTTCCTTTTATTCATGTGGGAAA TCAAGTAGTGTCAGAACTTGGCCCAATAGTCCAATTTGTTA AAGCCAAGGGCCATTCTCTTAGTGATGGGTTGGATGAAGT CCAAAAAGCAGAAATGAAAGCCTACATGGAATTAGTCAAC AATATGCTGCTGACTGCAGAGCTGTATCTCCAGTGGTGTGA TGAAGCTACAGTAGGGGAGATCACTATTGCTAGGTATGGAT CTCCTTACCCTTGGCCTCTGAGACTGGGAACACAACCATA CTTCTTCAATAAGCAACCTACTGAACTTGACGCTCTGGTTT TTGGCCATTTGTATACCATTCTTACCACACAATTGACCAGC GATGAACTTTCTGAGAAGGTGAAAAACTATAGCAACCTCC TGGCTTTCTGTAGAAGAATTGAACAGCACTACTTTGAAGA CTGGGGTAAAGGCAGGTTGTCT
**Mouse_*Mtx2* (p.41-161del) CDS**	GCCACCATGTCTCTGGTGGCGGAAGCGTTCGTCTCCCAGA TCGCAGCTACAGAACCTTGGCCTGAAAATGCGACCTTGTA TCAGCAACTGAGAGGGGAGCAAATTTTGCTTTCTGACAAT GCAGCACAGTGGGAAGTGAAACGTAAGATGAAAGCTATTG GATGGGGTAACAAGACTCTGGACCAGGTCTTAGAAGATGT AGACCAGTGCTGTCAAGCCCTTTCCCAGAGACTGGGAACA

CAACCATACTTCTTCAATAAGCAACCTACTGAACTTGACGC

TCTGGTTTTTGGCCATTTGTATACCATTCTTACCACACAATT

GACCAGCGATGAACTTTCTGAGAAGGTGAAAAACTATAGC

AACCTCCTGGCTTTCTGTAGAAGAATTGAACAGCACTACT

TTGAAGACTGGGGTAAAGGCAGGTTGTCT

Table S7: Sequences of Primers used in RT-PCR

Gene symbol	Forward 5’ to 3’	Reverse 5’ to 3’
Cpt1b	TACACGCATCCCAGGCAAAG	CGAGCCCTCATAGAGCCAAAC
Acsl1	TTCGCAGTGGCATCGTCAG	TGTGATCATCAGCCGGACTTTC
Acadm	GCTACAAGGTCCTGAGAAGTG	CTCCGTCAACTCGAAGCTAAA
Fabp3	TAGCATGACCAAGCCGACCA	ACCAGTTTGCCTCCGTCCAG
Pdha1	TCAATGCACATGTACGCCAAGA	TTATACTTGCAGGCCAGAGCAATTC
Pdhb	CACATCACTGTAGTTGCCCATTC	ATAGCTTCAATGTCCATTGGTCTG
Ogdh	CTGGCCAGGGTATCGTGTATGAG	CATCCGAGGGTCTGTGGTGA
Cs	TGGCCCAACGTAGATGCTCA	AGCCTAGGGCTCTGCTCCAGATA
Pfkm	GGATATGATACCAGGGTCACTGTTC	AAAGTGCCATCACTGCTTCCA
Pdk4	TGAACCAGCACATCCTCATATT	CTTCGACTACTGCTACCACATC
Idh2	GGAGAAGCCGGTAGTGGAGAT	GGTCTGGTCACGGTTTGGAA
Aldoa	CGTGTGAATCCCTGCATTGG	CAGCCCCTGGGTAGTTGTC
Eno3	CACAGCCAAGGGTCGATTCC	CCCAGGTATCGTGCTTTGTCT
Ldhb	CATTGCGTCCGTTGCAGATG	GGAGGAACAAGCTCCCGTG
Hadhb	ACTACATCAAAATGGGCTCTCAG	AGCAGAAATGGAATGCGGACC

Supplementary References

1. Li Y, Xiong Z, Jiang Y, Zhou H, Yi L, Hu Y, et al. Klf4 deficiency exacerbates myocardial ischemia/reperfusion injury in mice via enhancing ROCK1/DRP1 pathway-dependent mitochondrial fission. J Mol Cell Cardiol. 2023; 174: 115-32.
2. Li Y, Xiong Z, Yan W, Gao E, Cheng H, Wu G, et al. Branched chain amino acids exacerbate myocardial ischemia/reperfusion vulnerability via enhancing GCN2/ATF6/PPAR-α pathway-dependent fatty acid oxidation. Theranostics. 2020; 10: 5623-40.
3. Zhu P, Hu S, Jin Q, Li D, Tian F, Toan S, et al. Ripk3 promotes ER stress-induced necroptosis in cardiac IR injury: a mechanism involving calcium overload/XO/ROS/mPTP pathway. Redox Biol. 2018; 16: 157-68.
4. Zhou H, Zhang Y, Hu S, Shi C, Zhu P, Ma Q, et al. Melatonin protects cardiac microvasculature against ischemia/reperfusion injury via suppression of mitochondrial fission-VDAC1-HK2-mPTP-mitophagy axis. J Pineal Res. 2017; 63.
5. Xiong Z, Li Y, Zhao Z, Zhang Y, Man W, Lin J, et al. Mst1 knockdown alleviates cardiac lipotoxicity and inhibits the development of diabetic cardiomyopathy in db/db mice. Biochim Biophys Acta Mol Basis Dis. 2020; 1866: 165806.
6. Zhou H, Li D, Zhu P, Ma Q, Toan S, Wang J, et al. Inhibitory effect of melatonin on necroptosis via repressing the Ripk3-PGAM5-CypD-mPTP pathway attenuates cardiac microvascular ischemia-reperfusion injury. J Pineal Res. 2018; 65: e12503.
